# The Content of the 14 Metals in Cancellous and Cortical Bone of the Hip Joint Affected by Osteoarthritis

**DOI:** 10.1155/2015/815648

**Published:** 2015-08-17

**Authors:** Anetta Zioła-Frankowska, Łukasz Kubaszewski, Mikołaj Dąbrowski, Artur Kowalski, Piotr Rogala, Wojciech Strzyżewski, Wojciech Łabędź, Ryszard Uklejewski, Karel Novotny, Viktor Kanicky, Marcin Frankowski

**Affiliations:** ^1^Department of Analytical Chemistry, Faculty of Chemistry, Adam Mickiewicz University in Poznań, 61-614 Poznań, Poland; ^2^Department of Orthopedic and Traumatology, W. Dega University Hospital, Poznan University of Medical Sciences, 61-545 Poznań, Poland; ^3^Department of Water and Soil Analysis, Faculty of Chemistry, Adam Mickiewicz University in Poznań, 61-614 Poznań, Poland; ^4^Department of Spondyloorthopaedics and Biomechanics of the Spine, W. Dega University Hospital, Poznan University of Medical Sciences, 61-545 Poznań, Poland; ^5^Laboratory of Biomaterials and Peri-Implant Bioprocesses Engineering, Department of Process Engineering, Institute of Technology and Chemical Engineering, Poznan University of Technology, 60-965 Poznań, Poland; ^6^Department of Medical Bioengineering Fundamentals, Institute of Technology, Casimir the Great University, 85-064 Bydgoszcz, Poland; ^7^Department of Chemistry, Faculty of Science, Masaryk University, 611 37 Brno, Czech Republic

## Abstract

The aim of the study was to determine the content of particular elements Ca, Mg, P, Na, K, Zn, Cu, Fe, Mo, Cr, Ni, Ba, Sr, and Pb in the proximal femur bone tissue (cancellous and cortical bone) of 96 patients undergoing total hip replacement for osteoarthritis using ICP-AES and FAAS analytical techniques. The interdependencies among these elements and their correlations depended on factors including age, gender, place of residence, tobacco consumption, alcohol consumption, exposure to environmental pollution, physical activity, and type of degenerative change which were examined by statistical and chemometric methods. The factors that exerted the greatest influence on the elements in the femoral head and neck were tobacco smoking (higher Cr and Ni content in smokers), alcohol consumption (higher concentrations of Ni, Cu in people who consume alcohol), and gender (higher Cu, Zn, and Ni concentrations in men). The factors influencing Pb accumulation in bone tissue were tobacco, alcohol, gender, and age. In primary and secondary osteoarthritis of the hip, the content and interactions of elements are different (mainly those of Fe and Pb). There were no significant differences in the concentrations of elements in the femoral head and neck that could be attributed to residence or physical activity.

## 1. Introduction

Contaminated food, water, and air from industrial areas are the primary sources of metals in the human body [1]. Modern toxicology is dominated by issues related to exposure to chronic poisoning, which is associated with high levels of pollution [2]. Significant amounts of metals in the environment are the result of human activities; these metals participate in the metabolic processes of the body and pose serious threats to human and animal health. Interactions between elements may be either synergistic or antagonistic, causing deviations from their optimal range and resulting in secondary deficiency or toxicity [3, 4]. Bone tissue that exhibits slow remodelling reflects long-term exposure to metals and this can be used to indirectly assess the exposure to metals from the environment [5, 6]. Cancellous bone metabolism is more active than cortical bone and depends on many factors such as age, diet, and health status [7]. Many elements have a significant impact on bone metabolism [6]. The literature includes many reports on the content and relationships of elements in bone; however they mainly focus on other types of bone such as the tibia, patella, ribs, vertebrae, or the bones of the skull [6, 8].

*The Role of Selected Metals in the Human Body*. Zinc in cartilage and bone plays a role in growth and maturation and is involved in the hormonal regulation of intracellular signalling [9]. It has been proven that Zn deficiency leads to linear bone growth retardation, which is associated with dysfunction of growth hormone and insulin-like growth factor (IGF) dependent on the micronutrient [10]. Also, Zn has a powerful stimulating effect on osteoblasts, bone formation, and bone resorption by inhibiting the action of osteoclasts [11]. The expression of proteins that include Zn or play a role in its regulation, found in many cells of the connective tissue including osteoblast and osteocyte precursors, suggests an important role of the metal in the growth and maturation of bone [12]. Copper is a cofactor for lysyl oxidase, the enzyme responsible for the cross-linking of collagen fibres; disorders in the formation of crosslinks lead to a weakening of bone [13]. Copper deficiency leads to bone malformations during development, an increased risk of osteoporosis in the elderly. Some genetic diseases as Menkes syndrome and Wilson's disease are associated with severe Cu deficiency and severe Cu toxicity, respectively; they involve processes in the bone that involve Cu (osteomalacia, osteoporosis, and chondropathy) [14]. Copper also reduces the suppression of bone turnover by osteoblasts and osteoclasts [15]. An important role of Fe is its involvement in the formation of reactive oxygen species, which at low concentrations meets physiological demands, but, at higher concentrations, it is toxic to cells, causing their destruction [4, 16, 17]. The role of Fe in the bone tissue is not well understood. Iron is involved in the synthesis of collagen and the conversion of 25-hydroxy vitamin D to its active form. The Fe is necessary for the proper functioning of osteoblasts and excess Fe in osteoblasts may lead to metabolic bone disorders (osteoporosis, osteopenia, and osteomalacia) [13]. Strontium accumulates in bones and teeth (99%), but its effect on these tissues is not completely understood [18]. It has been shown that the effect of Sr is dose dependent. Low doses can stimulate bone formation (by stimulating the differentiation of osteoblasts) and increase bone density (which increases the synthesis of collagen and non-collagenous proteins). It increases the rate of bone mineralization and strength (often replacing calcium in the structure), the extension of the crystal lattice, and the solubility of crystalline embryos and decreases the rate of resorption (inhibiting osteoclast differentiation) [16, 19]. High doses of Sr may cause impaired mineralization and bone deformities due to abnormal calcium and phosphate metabolism [16]. Molybdenum in the human body accumulates in the liver, kidneys, bones, and teeth [20] An excess of Mo is toxic and causes bone deformities similar to the changes occurring in the rheumatoid arthritis, tooth decay, and disorders of lipid and protein [4]. Chromium builds stable complexes with proteins and has the ability to precipitate proteins, resulting in negative effects on the skin and mucous membranes. Allergy to chromium compounds is a common cause of eczema, characterized by oedema of the eyelids, blushing nettle, oedematous papules, and exudate on the face, neck, forearms, arms, hands, and fingers [4]. The role of nickel in the physiology of the human body is not well understood. In humans, Ni deficiency causes a decrease in oxygen consumption and an increase in the accumulation of liver fats. This occurs mainly in soft tissues, although its presence and influence in bone metabolism have also been confirmed [4, 16, 21, 22]. Barium is not metabolized by the body, but it can be transported or metabolically incorporated into tissue complexes. Toxic effects are associated with impaired digestive and respiratory systems, as well as the inhibition of bone mineralization [16]. Lead has the ability to accumulate in the body, mainly in the liver, brain, kidneys, muscles, and bones. Most (90–95%) Pb accumulates in the bone tissue, where it is deposited in an exchange of Ca^2+^ ions in biological processes mainly in the less metabolically active cortical bone. Resorbed Pb is present in the body in two pools: quick change (blood, soft tissue), where it produces acute effects and slow change (bone), which is metabolically inactive. Pb leads to a decrease in bone mineral density, which is manifested by a greater susceptibility to fractures and bone healing disorders. This is done by altering the metabolism of osteoblasts and osteoclasts, reducing the production of stem cells, osteoblasts, and disorders of calcium homeostasis. The effects on bone cells may be direct (decrease activity of osteoclasts and osteoblasts) or indirect (disturbances in the metabolism of 1.25-dihydroxyvitamin D and parathyroid hormone (PTH)). Lead also competes with the metal binding sites on enzymes, which are cofactors, with zinc in porphobilinogen synthesis or proteins, and with iron in the transfer [23, 24].

The main aim of this study was to determine the concentration of Ca, Mg, P, Na, K, Zn, Cu, Fe, Mo, Cr, Ni, Ba, Sr, and Pb in the proximal femoral head (cancellous bone) and femoral neck (cortical bone) of the hip joint affected by osteoarthritis. The present study was conducted to assess differences between the concentrations of metals in the femoral head and neck, according to sex, and also to examine, by statistical and chemometric analysis, a possible correlation between various factors, including: age, place of residence, tobacco consumption, alcohol consumption, contact with chemicals, and the content of elements in the femoral bone. The flame atomic absorption spectrometry (FAAS) and inductively coupled plasma emission spectrometry (ICP-AES) analytical techniques were applied for the determination of elemental metals in bone samples.

## 2. Materials and Methods

### 2.1. Ethics Statement

The use of femoral heads in the investigations was permitted by the Bioethical Committee of the University of Medical Sciences in Poznan (Poland) (Permit number: 172/4) and all patients provided written informed consent prior to participation.

### 2.2. Patients

The sample consisted of 96 patients who were operated on for total hip replacement (THR). All patients lived in the Wielkopolska region of Poland. A history of the disease did not affect the outcome of the study. There is no major industry concentration in this region.  Table 1 shows the characteristics of patients enrolled in the study, with particular emphasis on factors that possibly affect the concentration of selected metals in the femoral bone.

### 2.3. Characterization and Sampling of Femoral Bone Samples

All of the femoral heads were acquired intraoperatively from the patients undergoing total hip arthroplasty for osteoarthritis of the hip. In the total hip replacement procedure, a metal ball replaces the worn head of the thigh bone and a cup (often plastic) replaces the worn socket. The shape of the examined proximal ends of the femurs had been changed by the degenerative process. Femoral heads were flattened and bony excrescences could be observed at the head/neck border. Bone tissue condensation at the superolateral part of the femoral head and cysts in femoral head and neck were present. Articular surfaces exhibited defects that were accentuated at the loading area. Articular cartilage was pathologically changed, softened, and disintegrated, with areas of local destruction due to subchondral congestion and blood vessel infiltration. Directly after acquisition, the spongiest bone was separated from the femoral heads under sterile conditions. Samples were cut from the head and neck of the femur. In the case of the femoral neck, samples were collected with a patch section thickness of 1-2 mm and the 5 mm slice was taken in the shape of a triangle.

### 2.4. Bone Preparation and Determination the Elemental Metals in Samples

The frozen bone samples were freeze-dried using a Lyovac lyophilizer GT2e (Steris, Germany) for 24 hours. After drying, approximately 0.5 g of the sample was weighed and placed in a Teflon bomb Mars 5 Xpress microwave oven (CEM, USA). A 10 mL volume of suprapure nitric acid (V) (Merck, Germany) was added. The prepared samples were allowed to stand for 8 hours to slow mineralization. The samples were then mineralized in a microwave oven using a modified EPA method 3051. After cooling, the samples were placed into flasks and filled to 50 mL with demineralized water. The concentrations of Mo, Cr, Zn, Pb, Cu, Ni, Fe, Mg, P, and Ca were determined using ICP-AES Jobin Yvon, 170Ultrace (Jobin Yvon, France) with laterally viewed plasma. The samples were nebulized using a concentric Meinhard nebulizer. The concentrations of Na and K were determined using the Shimadzu AA-7000 Flame AAS analytical technique (Shimadzu, Japan). The accuracy of the procedure was verified by analysis of NIST CRM 1400 (Bone Ash) by both analytical techniques. The recoveries for analysed elements varied from 94.6% to 109%. The limits of detection (LOD) are as follows: Mo < 0.36 mg/kg; Cr < 0.24 mg/kg; Zn < 0.2 mg/kg; Pb < 0.63 mg/kg; Cu < 0.2 mg/kg; Ni < 0.6 mg/kg; Fe < 0.3 mg/kg; Mg < 0.1 mg/kg; P < 1.0 mg/kg; Ca < 0.1 mg/kg; Na < 0.04 mg/kg; K < 0.6 mg/kg. For statistical and chemometrics analysis, a half value of LOD was used.

### 2.5. Statistical and Chemometrics Analysis

The analysis used Statistica 7.0 (StatSoft) software. To determine compliance with the expected normal distribution of results, we used a Shapiro-Wilk test (*p* < 0.05). To compare the impact of various environmental factors on the concentration of analysed metals in the bone we used a Kruskal-Wallis test, and, in the case of significant differences, a Mann-Whitney *U*-test was performed (*p* < 0.05). In addition, we determined the Spearman's rank correlation between trace elements occurring in the materials and between the different studied metals in different parts of the hip joint (cortical bone, neck, cancellous bone, head). Principal Component Analysis was used to calculate significant explanatory factor scores for each lake using all physical and chemical variables independently. The environmental factor scores, biomass estimates, and biological variables were compared using correlation analysis.

## 3. Results and Discussion

It is becoming increasingly important to assess the risk to bone tissue from exposure to metals of environmental and occupational origin [25]. The processes of bone remodelling are active throughout the lifespan and therefore can be an indicator of metal accumulation in bone tissue from long-term chronic exposure. The toxic effects may be revealed after many years of exposure or may appear suddenly. Metals may replace other elements necessary for normal metabolism, disrupting a number of processes that depend on the internal equilibrium system [26, 27]. The largest number of essential correlations between the studied elements occurred in the trabecular bone, followed by cortical bone, and the least number was in the articular cartilage [28]. The trace elements are concentrated in some tissues and organs; their distribution depends on their condition, age, and individual hereditary properties [29, 30].

The results of the content of the 14 elements in samples of the cancellous and cortical bone of the femur are presented in Table 2. In addition, for the examined group of elements, we verified the type of distribution, using the Shapiro-Wilk test. For each element, we also applied the Mann-Whitney *U*-test to determine the significance of difference in the concentrations of elements between the femoral neck and head.

In the case of calcium, the content (as a median value) in the femoral head and neck was 130 g/kg and 153 g/kg, respectively. The obtained concentrations of calcium were much higher in comparison to the report of Kuo et al. [29], in which the concentration of Ca was 82 g/kg in cancellous bone. The phosphorus content in the femoral head and neck was 59 g/kg and 69 g/kg, respectively. The mean content of phosphorus in cancellous bone was comparable to those obtained by Zaichick [31], where the concentration of P was 56 g/kg. The concentration of phosphorus in cortical bone was much lower in comparison with Zaichick [31], where the concentration of P was 107 g/kg. The content of magnesium in the femoral head and neck was 1381.2 mg/kg and 1595.3 mg/kg, respectively. The mean content of magnesium was compared to that obtained by Brodziak-Dopierała et al. [28], where the concentration of Mg was 1650.85 mg/kg and 1376.14 mg/kg, respectively. In our study, the content of Mg was higher in cortical bones than in cancellous bone. The median Na and K contents in the femoral head and neck were 5428.6 mg/kg, 4662.1 mg/kg and 679.3 mg/kg, 647.1 mg/kg, respectively. These were comparable to those reported by Brodziak-Dopierała et al. [28], where the concentrations of Na were 4745.47 mg/kg and 6790.35 mg/kg, respectively, and the concentrations of K were 828.80 mg/kg and 767.11 mg/kg, respectively, in the femoral head and neck. Regarding Zn, Kuo et al. [29] and Darrah [32] found that the content of metal is higher than 100 mg/kg, while in the Polish studies the content of metal was lower (61.48–94.72 mg/kg) [28, 33, 34]. In our research, the content of Zn was approximately 70 mg/kg. The median copper content in the femoral head and neck was 0.83 mg/kg and 0.72 mg/kg, respectively. The mean concentration of copper in the femur was comparable to the results obtained in cancellous bone by Lanocha et al. [33] 0.67 mg/kg and Jurkiewicz et al. [34] 0.79 mg/kg. The median iron content in the femur was 91 mg/kg, which was much higher in comparison with Lanocha et al. [33] (cancellous bone 43.9 mg/kg and cortical bone 40 mg/kg) and Jurkiewicz et al. [34] (47.6 mg/kg) and much lower in comparison with Darrah [32] (123.35 mg/kg and 108.15 mg/kg, resp.). In this study, the median chromium content in the femoral head and neck was 0.49 mg/kg and 0.83 mg/kg, respectively. The median concentration of Cr was 2 times higher in the femoral head in comparison with Darrah [32] (0.812 mg/kg) and 10–20 times lower in the femoral head and neck in comparison with Brodziak-Dopierała et al. [28] (10.42 mg/kg and 14.99 mg/kg, resp.). The median barium content in the femoral head and neck was 2.05 mg/kg and 2.25 mg/kg, respectively. The median concentration of Ba was slightly lower in comparison with Darrah [32] 3.045 mg/kg and 2.595 mg/kg. The median Mo content in the femoral head and neck was 0.18 mg/kg. The median concentration of molybdenum was 10 times higher in comparison with Darrah [32] (0.013 mg/kg and 0.019 mg/kg, resp.). The mean Ni content in the femoral head and neck was 0.6 mg/kg and 0.79 mg/kg, respectively. The mean concentration of nickel was 10 times lower in comparison to Brodziak-Dopierała et al. [28] in the cancellous and cortical bone (4.56 mg/kg and 6.86 mg/kg, resp.). The mean content of toxic lead in the femoral head and neck was 1.15 mg/kg and 1.08 mg/kg, respectively. The mean concentration of Pb was 6 and 12 times lower in comparison to Brodziak-Dopierała et al. [28] in the cancellous and cortical bone (6.22 mg/kg and 12.27 mg/kg, resp.). The contents of Pb obtained by Lanocha et al. [33] were 2 times lower (0.5 mg/kg).

### 3.1. Factors Influencing Metal Concentrations in Cancellous and Cortical Bone

It is well known that interactions between elements can cause disequilibrium in their optimal range of concentrations and, consequently, secondary deficiency or toxicity [28]. The results of this study used various statistical tools and specified the influence of different environmental factors on the occurrence and content of 14 elements in the femoral bone. Distributions of macroelement concentrations (Ca, P, Mg, and Na) in the femoral neck had the characteristics of a normal distribution and in the case of femoral head the same type of distribution was observed for Mg and Na, which was confirmed by the Shapiro-Wilk test. We found that, in the femoral neck, macroelement concentrations (Ca, P, and Mg) and two trace element concentrations (Sr, Cr) were significantly higher than those in femoral head (Table 2). The Spearman's rank correlation allowed us to determine the relationships between examined metals in the bones of the patients (Table 3).

Strong and very strong correlations were found between Ca, P, Mg, Na, and Zn for the femoral head and neck. A moderate correlation was found between two metals, Sr and Ba, and between them and the Ca, P, Mg, Na, and Zn for the femoral head and neck. Other correlations for the femoral head and neck were Fe/Cr, Cu/Ni, and Cu/Zn, those only for the femoral neck were Cu/Fe and Mo/Pb, and only for femoral head were Mo and Cu. Weak correlations were found between Pb and the macroelements Ca, P, Mg, and Na for the femoral neck.

**Figure 1:**
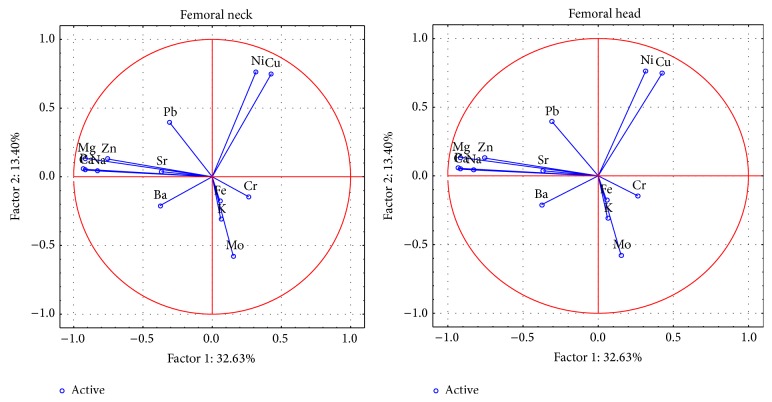
A graphic illustration of Principal Components Analysis. Projection of the variables on the factor plane of the first two principal components for femoral head and neck for all samples.

The changes between the femoral head and neck were analysed by PCA chemometric technique. For the 6 important components, the total variance was 79.1% in the femoral neck and 77.4% in the femoral head (Figure 1).

In femoral neck and head, the first component represents the change of the contents of Ca, P, Na, Mg, and Zn. In the femoral head, only the first component indicates the meaning of the Ba. The second factor describes the changes of content of Cu, Ni. In case of Cr, the second component for the femoral head and the third component for the femoral neck concerns the role of element content. In the case of Fe content, the meaning is indicated by the third component in both parts. The meaning of Ba content is indicated by the fifth component in the femoral neck. The fourth and fifth components for the femoral head and the sixth component for the femoral neck concern the role of Pb content.

#### 3.1.1. Sex

To determine the differences in the content of various elements in the femur samples between women and men, the Mann-Whitney test was used (Table 4). The differences in 4 metal contents in the femoral heads of women were statistically significant (*p* < 0.05) from those of men. Higher contents of Cu, Ni, Zn, and Pb were found in the femoral heads of men than in women. Brodziak-Dopierała et al. [13, 22] found that the content of Ni and Fe in the femoral head in men was higher than in women. The present study supports these findings. S. Zaichick and V. Zaichick [35] found higher Ca, K, Mg, Na, and Sr mass fractions in female femoral necks compared with male femoral necks, while Budis et al. [18] found that the concentrations of Fe and Sr were similar in men and women. In our study, more correlations were observed for men than for women.

Comparisons between male and female femoral necks showed negative correlations for Cu/Zn, Mo/Pb, and Cu/Mo and positive correlations for Cr/Ni and Cr/Fe. The comparison between the femoral necks of women and men showed positive correlations of Pb to Ca, P, Mg, and Na only for the men. The greatest divergence of the correlation coefficient in the head of the femur between women and men was observed in the case of Na. All correlations between metals according to the sex in the femoral neck and head are presented in Tables 5 and 6.

**Figure 2:**
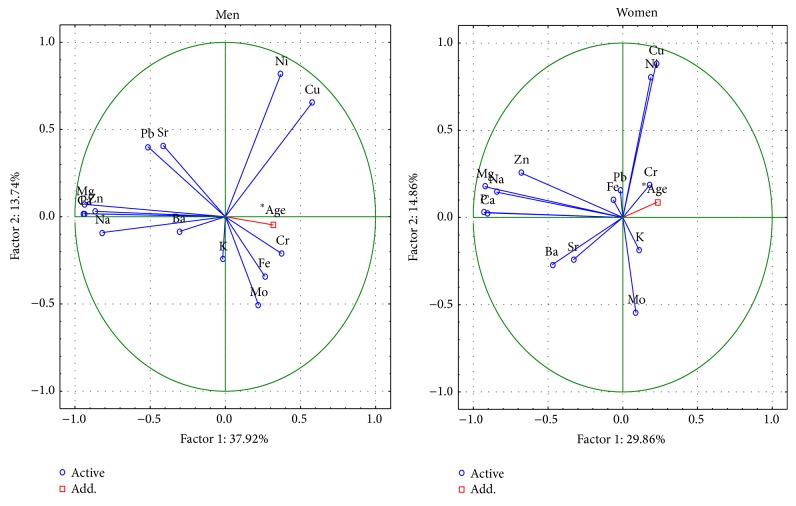
A graphic illustration of Principal Components Analysis of contents of elements according to sex for femoral neck. Projection of the variables on the factor plane of the first two principal components for men and women.

**Figure 3:**
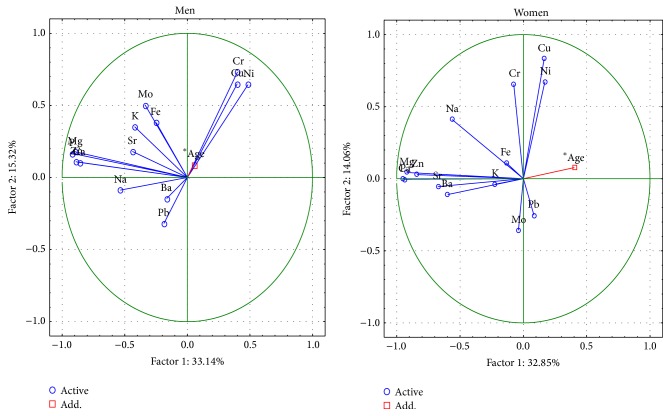
A graphic illustration of Principal Components Analysis of contents of elements according to sex for femoral head. Projection of the variables on the factor plane of the first two principal components for men and women.

Conducting the PCA showed that the changes between women and men are described by 6 important components, the total variance of which is 79.1% in the femoral neck and 77.4% in the femoral head (Figures 2-3).

In the femoral neck and head, the first component represents the change of the contents of Ca, P, Na, Mg, and Zn. In the femoral neck, the first component indicates the meaning of the Pb only in men. The second factor defines the changes of content of Cu, Ni, Mo, and Cr in women as well as in men. The meaning of Ba content in the femoral neck and head is indicated by the third component only in men. The third component for women and men concerns the role of Fe content. The meaning of K was represented by the fourth component for women and the fifth component for men. The fourth component for men and the fifth component in both concern the role of Pb content in the femoral head. The meaning of K was represented by the fifth and sixth components for women and the third component for men in the femoral head. The comparison with the PCA conducted by Brodziak-Dopierała et al. [27] showed that the first component of their data was represented by the change of the contents of Cr, Ni, Zn, Na, Ca, and Cu, and, in the second order, there are changes of content of Mn, Cd, Pb, and Fe, regardless of gender. It is important to note that only the fifth component concerns the role of Mg content. The present data [27] are contrary to this study in which the first factor described structural elements.

It should be emphasized that the results proved that sex is a major factor that determines the type of interaction of metals with elements of physiological importance [36]. According to the literature, the majority of trace elements are not significantly different between the bones of male and female patients, with the exception of Zn in trabecular tissues and Pb in cortical tissues of men [37, 38]. This statement contradicts our findings, which showed apparent differences in the femoral head for Zn, Pb, Cu, and Ni for both genders. Brodziak-Dopierała et al. [28] found in both men and women statistically significant correlations between the concentrations of Cr-Ni, Cr-Na, Ni-Cd, and Ni-Cu, and in Zn-Na, Ca-Ni, and Cu-Zn correlations were observed only in the male patients. In our study, it was observed that Zn accumulated in femoral neck of men, simultaneously with Pb. Taking into account that Zn may reduce the toxic effects of Pb [32, 39], the results may indicate a low risk of toxic influence of Pb among men.

#### 3.1.2. Smokers

Analysis with the Mann-Whitney *U*-test showed a statistically significant difference between smokers and nonsmokers. The concentrations of Ca, Mg, and P in the cancellous bone were significantly lower in smokers than in nonsmokers. Of the trace elements, Zn concentration was lower in smokers (of borderline significance). Pb and Cr were found in higher concentrations in smokers (difference not significant). There were no significant differences in the concentrations of metals in the femoral neck between smokers and nonsmokers (Table 7).

In several previous studies, the differences in the concentrations of some metals in the femur between smokers and nonsmokers were demonstrated [40, 41]. Only in nonsmokers' femoral necks we did find a significant moderate positive correlation between Pb and Zn Ca, P, Mg, and Na and a negative correlation for Pb/Mo. The femoral neck had a significant high correlation for Cr/Ni in smokers that was absent in nonsmokers. The metals Ca, P, and Mg moderately negatively correlated with Fe in nonsmokers, while smokers' correlation coefficient was close to zero. In nonsmokers, we observed weak negative correlations for Mo/Cu and Ni/K, and, in smokers, we observed correlations for Mo/Cu and Na/Pb (Tables 8-9).

Brodziak-Dopierała et al. [28] found correlations between Cr-Cu, Cr-Zn, Cu-Zn, and Cu-Na in smokers that did not occur in nonsmokers. The correlations between Cr-Ni, Ni-Cu, and Ni-Zn were found in both smokers and nonsmokers [28]. Budis et al. [18] found no statistically significant differences in the concentrations of Fe and Sr between the group of smokers and nonsmokers. In smokers, a positive correlation between the levels of Ni and Cr that was not present in nonsmokers was found. Additionally, in smokers, they observed a significantly increased level of Pb in bone tissue [22].

**Figure 4:**
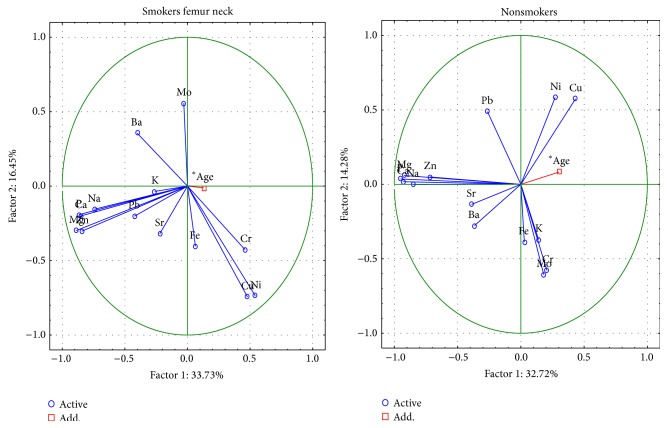
A graphic illustration of Principal Components Analysis of contents of elements according to cigarette smoking for femoral neck. Projection of the variables on the factor plane of the first two principal components for smokers and nonsmokers.

The changes in the femoral neck between smoking and nonsmoking people are described by 6 important components, the total variance of which is 79.1% (Figure 4). The first component indicates, as above, the presence of structural elements Ca, P, Na, Mg and Zn in the structure of hydroxyapatite. The meaning of Mo, Cr, Cu and Ni in ion exchange is characterized by the second component, and in nonsmokers also Pb. The Pb, Sr, and K in smokers and the Cu, Ni, and Fe in nonsmokers are represented by the third component.

**Figure 5:**
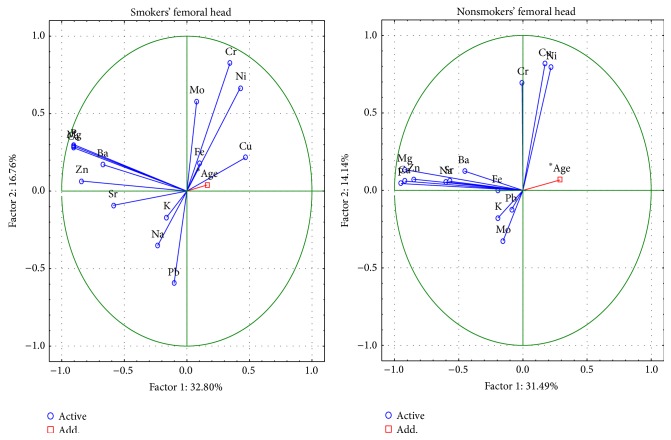
A graphic illustration of Principal Components Analysis of contents of elements according to cigarette smoking for femoral head. Projection of the variables on the factor plane of the first two principal components for smokers and nonsmokers.

The changes in the femoral head between smoking and nonsmoking people are described by 6 important components, the total variance of which is 77.6% (Figure 5). The first component indicates, as above, the presence of macroelements Ca, P, Na, Mg, and Zn in the structure of hydroxyapatite. The meaning of Cr and Ni in ion exchange is represented by the second component; it also includes Cu, in nonsmokers and Mo and Pb in smokers.

#### 3.1.3. Contact with Chemicals

To find the differences in the content of elements in the femur between patients with or without chemical contact, the Mann-Whitney test was used (Table 10). The concentration of Ni in the femoral head was significantly higher in subjects with exposure to environmental chemicals in comparison to those who had no contact with metals in the environment.

The relations between the examined metals in the bones of the patients were based on Spearman's rank correlation (Tables 11-12). A negative correlation was found between Ca and Pb for the femoral neck in patients having contact with chemicals and a positive correlation in patients having no contact with chemicals; only a positive correlation was found for the femoral head in patients having contact with chemicals.

**Figure 6:**
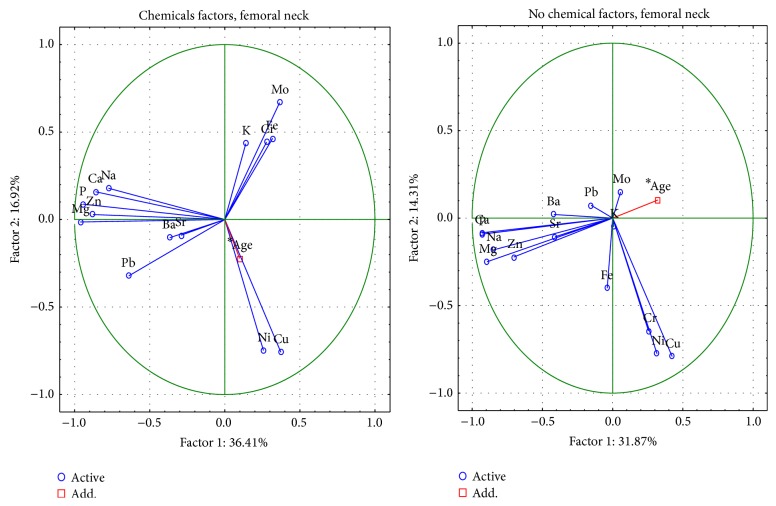
A graphic illustration of Principal Components Analysis of contents of elements according to chemical factors for femoral neck. Projection of the variables on the factor plane of the first two principal components for chemical and no chemical factors.

**Figure 7:**
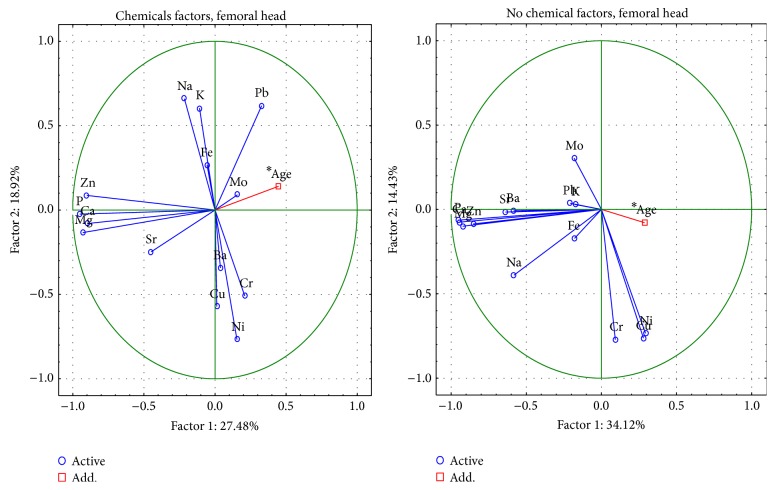
A graphic illustration of Principal Components Analysis of contents of elements according chemical to factors for femoral head. Projection of the variables on the factor plane of the first two principal components for chemical and no chemical factors.

The differences in metal physiology in the femoral head and neck between people with and without chemical contact were described by PCA analysis (Figures 6 and 7). The first component indicates the presence of Ca, P, Na, Mg, and Zn. The meaning of Cr, Cu, and Ni content in ion exchange is characterized by the second component, and, in people with chemical contact, also Pb, K, and Na. The Pb and Fe in non-chemical-contact patients and Mo, Cr, Fe, and K in patients with chemical contact are represented by the third component.

#### 3.1.4. Alcohol

Analysis with the Mann-Whitney *U*-test showed significant differences between alcohol drinkers and abstainers. Patients who consume alcohol had significantly higher mean Cu content compared to abstainers in both femoral heads and necks.

It should be emphasized that these results have not been previously identified by other researchers. Also the concentrations of Ni and Pb were significantly higher in the femoral heads of drinkers compared to abstainers (Table 13). Exposure to Pb or/and ethanol decreased bone formation and increased its resorption, resulting in the bone demineralization. These effects were accompanied by destroying the hormonal regulation of mineral metabolism and Ca and P imbalance [42]. The mechanism underlying ethanol-associated osteopenia seems to be a direct effect of alcohol on bone cells and an indirect or modulating effect through mineral regulating hormones such as vitamin D metabolites, parathyroid hormone, and calcitonin. The modulating effects of these hormones on bone and mineral metabolism are observed in acute and chronic alcohol consumption [43].

The correlations for metals in femoral necks and heads are described by the Spearman's rank correlation (Tables 14 and 15). In the femoral neck, we observed a statistically significant negative correlation between Fe and age and the concentration of metals Ca, P, and Mg in abstainers. The deposition of Pb with age was demonstrated in abstainers, but no correlation was observed in drinkers. In the femoral head, we observed a positive correlation between Mo/Fe in people who consume alcohol and negative one between Mo/Cu in abstainers. The drinkers had a significantly negative correlation for Ni/Zn and positive one for Ni/Cu. In both groups, Cr significantly correlated positively with Cu, and in people who drink alcohol it correlated negatively with Pb. Iron positively correlated with age in abstainers. The heads of the femur showed a statistically significant negative correlation between Cu and Ni and the concentration of metals (Ca, P, Mg, and Zn) in people who drink little or average amounts, while abstainers showed no such correlation.

**Figure 8:**
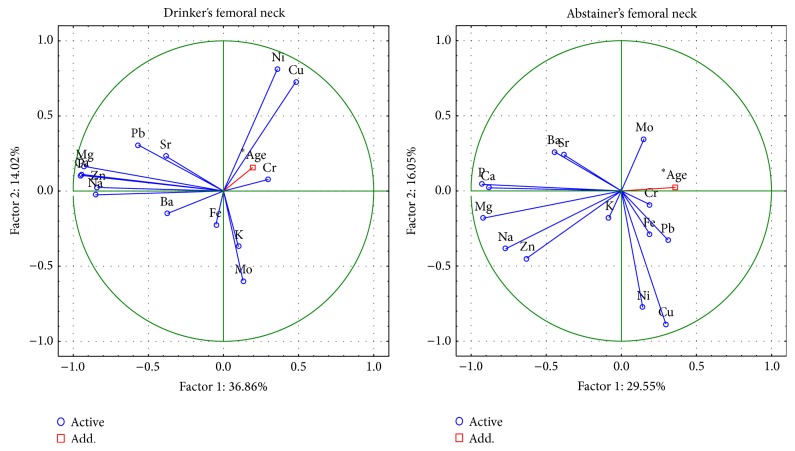
A graphic illustration of Principal Components Analysis of contents of elements according to alcohol consumption for femoral neck. Projection of the variables on the factor plane of the first two principal components for alcohol drinkers and abstainers.

The changes in the femoral head between drinkers and abstainers are described by 6 important components, the total variance of which is 77.6% (Figure 8). The first component indicates, as above, the presence of macroelements Ca, P, Na, Mg and Zn in the structure of hydroxyapatite and, in abstainers, Sr, Ba and Na. The Cu, Cr, and Ni are characterized by the second component and these metals are opposite in drinkers and abstainers. The Pb in drinkers is represented by the third component.

**Figure 9:**
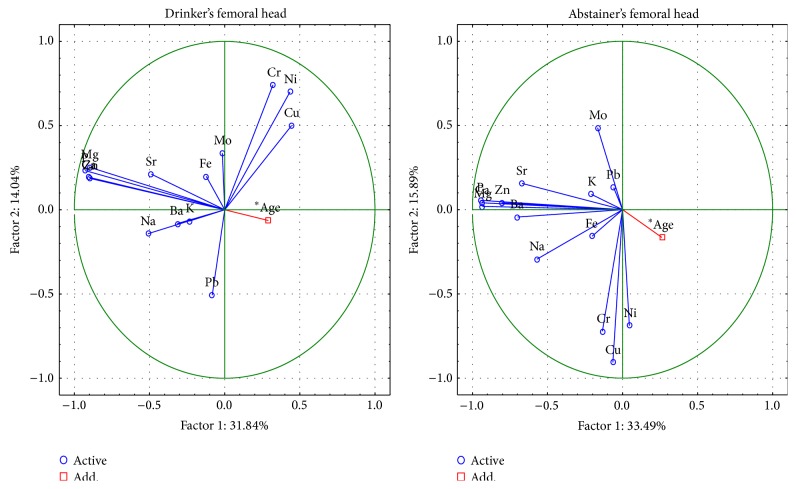
A graphic illustration of Principal Components Analysis of contents of elements according to alcohol consumption for femoral head. Projection of the variables on the factor plane of the first two principal components for alcohol drinkers and abstainers.

The changes in femoral neck between drinkers and abstainers are described by 6 important components, the total variance of which is 79.1% (Figure 9). The first component indicates, as above, the presence of Ca, P, Na, Mg, and Zn. Additionally, the first component indicates the content of Pb only in alcohol drinkers. Cu and Ni were characterized by the second component and Cr by the third component, but the values of these components were opposite in drinkers and in abstainers.

#### 3.1.5. Residence-Related Differences

The analysis with the Mann-Whitney *U*-test showed no statistically significant differences in the concentrations of the examined metals in the cortical and cancellous bone between patients living in villages and those living in cities (Table 16).

Previous studies described the existence of heavy metals in several regions of Poland. Jurkiewicz et al. [34] evaluated the similar content of Ca, P, Mg, P, Fe, Zn, Cu, Pb, Cd, As, and Ag in femoral heads of inhabitants of southern Poland (Silesia, Cracow) and middle Poland (Łódź). Specimens from different regions differed in Pb and Cd content, illustrating the differences in environmental pollution exposure [26]. Budis et al. [18] found no statistically significant residence-related differences in the concentrations of Sr, Mn, or Fe.

#### 3.1.6. Age

To determine the differences in the content of various elements in the femur samples in different age groups, the Kruskal-Wallis test was used. A marked tendency to decrease the concentration of structural elements with age has been shown by a statistical significance for femoral head Ca, P, and Mg. We also found significant reductions in the concentration of Sr with age in the femoral neck.

A statistically significant tendency for the Ca, Mg, and P content to decrease with age was found in the human rib bone, regardless of gender (Table 17). The mass fraction of Fe in the male rib bone increases with age. Higher Ca, Mg, Na, P, and Sr mass fractions and lower Fe content were typical of female ribs compared to male ribs [6, 44]. Previous research indicates that trace metal concentrations of Zn and Sr decrease with age [45]. Conversely, only Pb is shown to increase with age, which was previously attributed to higher Pb exposure in the past. Alternatively, the higher Pb concentrations may indicate additional exposure over time that exceeds endogenous release during bone remodeling, that is, an accumulation of Pb [32].

## 4. Conclusions

The standard deviations obtained for all trace elements are, respectively, large. This finding is attributable to the wide individual variation of trace element mass fractions in the human femoral bone affected by osteoarthritis. The factors that most affect the content and interactions of the elements in the femoral head and neck are smoking (increased content of chromium and nickel in smokers), alcohol consumption (higher concentration of nickel and copper in people consuming alcohol), and gender (higher concentrations of nickel, copper, and zinc in men). We confirmed significant differences in the content of metals between cancellous and cortical bone. The factors contributing to the toxic accumulation of lead in bone tissue are smoking, consumption of alcohol, male gender, and age. The concentrations of calcium, phosphorus, magnesium, and strontium decrease with age and the concentration of lead increases with age. Loss of elements, calcium, phosphorus, magnesium, zinc, sodium, and strontium from the femoral neck, progresses with age and is more common in city dwellers and those who are physically inactive. Radiological and clinical factors (pain, BMI, and cortical index) do not correlate significantly with the contents of the elements in the femur.

## Highlights

- We found a significantly higher content of Cu, Ni, and Pb in drinkers.
- In the femoral neck, Cr strongly positively correlated with Ni in smokers.
- We determined that cigarette smoking, alcohol consumption, and gender were the most influential factors.
- We observed much higher concentrations of Zn, Cu, Ni, and Pb in the femoral head in men.
- We identified a higher Ni content in people who had contact with chemicals.

## Figures and Tables

**Table 1:** Information on patients included in the study.

Factors	Sample of patients (*n* = 96) who were operated on for total hip replacement (THR)	
		AM ± SD/Min. and Max.

Age [years]	Women (*n* = 57)	64.5 ± 14.2/25–87
	Men (*n* = 39)	63.2 ± 10.2/42–91

Body weight [kg]	Women (*n* = 57)	69.8 ± 14.2/45–115
	Men (*n* = 39)	84 ± 16.1/45–114

Height [cm]	Women (*n* = 57)	159.9 ± 6.8/139–174
	Men (*n* = 39)	172.2 ± 6.4/155–185

Width of the femur [cm]	Women (*n* = 57)	2.98 ± 0.29/2.4–3.7
	Men (*n* = 39)	3.31 ± 0.3/2.5–3.8

		Number of patients (percent)

Place of residence	Village	24 (25%)
	City >10 000	16 (16.7%)
	Town <10 000	56 (58.3%)

Type of hip osteoarthritis	Primary, idiopathic	54 (56.3%)
	Secondary, developmental dysplasia of the hip	42 (43.8%)

Cigarette smoking	Nonsmoker	73 (76%)
	Irregular smoker	6 (6.3%)
	Regular smoker	17 (17.7%)

Alcohol drinking	Nondrinker	46 (47.9%)
	Occasionally	23 (24%)
	Often	27 (28.1%)

Physical activity	During puberty	60 (62.5%)
	Before the disease	30 (31.3%)
	Constant	23 (24%)

Environment pollution	Air	5 (5.2%)
	Water	3 (3.1%)
	Soil	2 (2.1%)

Contact with chemicals in the workplace	*Type of chemicals:* Fertilizers, plant protection arable, heavy metals, paints, adhesives, plaster, tannery articles, photographic chemicals, reagents used in metallurgy, other chemicals, and detergents	30 (31.3%)

**Table 2:** Concentrations of elements (in mg/kg on dry mass basis) and differences between them in the cancellous and cortical bone of the femur (*N* = 96).

Metal	Femoral head	Femoral neck	M-W
	AM ± SD	AM ± SD	
	Med.	Med.	
	Min. and Max.	Min. and Max.	

Ca	136705.6 ± 36168.7	157212.3 ± 40448.5	*p* < 0.01
	130519	153269	
	69364–216920	65524–252100	

P	62723 ± 16774.4	70652.3 ± 18279.3	*p* < 0.01
	59094.6	69616.2	
	33104–98424	29480–109180	

Mg	1446.76 ± 359.2	1585.8 ± 320.2	*p* < 0.01
	1381.2	1595.3	
	834.3–2528.6	782.9–2243.9	

Na	5466.5 ± 1043.9	4681.2 ± 884.6	*p* < 0.01
	5428.6	4662.1	
	3244.2–8566.9	2228.7–6624.7	

K	842.4 ± 685.7	972.1 ± 1136.3	NS
	679.3	647.1	
	297–6343.1	250–9340	

Zn	72.09 ± 16	68.7 ± 13.38	NS
	69.84	66.21	
	45.68–112.68	43.06–108.17	

Cu	0.91 ± 0.88	0.89 ± 1.15	NS
	0.83	0.72	
	0.04–3.56	0.04–6.58	

Cr	1.33 ± 2.24	1.44 ± 1.73	NS
	0.49	0.83	
	0.12–14.32	0.12–8.09	

Ni	0.6 ± 1.26	0.79 ± 2.28	NS
	0.03	0.03	
	0.03–7.38	0.03–14.88	

Fe	124.42 ± 106.18	131.52 ± 139.28	NS
	91.14	90.97	
	10.5–499.28	20.63–839.58	

Sr	44 ± 26.5	47.3 ± 22.7	*p* = 0.045
	36.6	43.2	
	1.3–150.9	16.3–137.5	

Ba	2.47 ± 1.68	2.41 ± 1.3	NS
	2.09	2.25	
	0.13–9.05	0.24–7.01	

Mo	0.56 ± 0.6	0.73 ± 0.68	*p* = 0.034
	0.18	0.18	
	0.18–2.04	0.18–3.1	

Pb	1.15 ± 1.51	1.08 ± 1.37	NS
	0.32	0.32	
	0.32–6.28	0.32–7.52	

AM: arithmetic mean; SD: standard deviation; Med.: median; Min.: minimal obtained concentration, Max.: maximum obtained concentration; M-W: Mann-Whitney *U*-test; *p*: level of significance; NS: nonsignificant difference.

**Table 3:** Spearman correlation coefficients for metals found in femoral neck and head for all samples.

	Mo	Cr	Zn	Pb	Cu	Ni	Fe	Mg	K	P	Sr	Ba	Na	Ca	Age
	Head														
Mo	x	−0.08	0.09	0.02	−0.22^*∗*^	−0.10	0.05	0.07	0.07	0.08	0.04	−0.01	−0.13	0.03	0.02
Cr	0.11	x	−0.03	−0.16	0.43^*∗*^	0.16	0.34^*∗*^	0.07	0.19	0.05	−0.09	0.06	0.13	0.02	0.03
Zn	−0.13	−0.07	x	0.08	−0.09	−0.13	0.14	0.80^*∗*^	0.11	0.81^*∗*^	0.52^*∗*^	0.40^*∗*^	0.46^*∗*^	0.79^*∗*^	−0.11
Pb	−0.32^*∗*^	−0.18	0.26^*∗*^	x	−0.01	−0.02	0.03	0.06	0.02	0.01	−0.05	0.06	0.20	0.05	0.10
Cu	−0.17	0.27^*∗*^	−0.12	0.12	x	0.44^*∗*^	0.15	−0.05	0.21^*∗*^	−0.14	−0.08	−0.13	0.03	−0.14	0.04
Ni	−0.03	0.31^*∗*^	−0.16	0.01	0.57^*∗*^	x	−0.18	−0.14	−0.29^*∗*^	−0.14	−0.07	0.04	−0.20	−0.15	0.02
Fe	−0.02	0.37^*∗*^	−0.18	−0.12	0.34^*∗*^	0.14	x	0.05	0.30^*∗*^	0.09	−0.03	−0.03	0.12	0.04	0.13
Mg	−0.16	−0.09	0.74^*∗*^	0.27^*∗*^	−0.25^*∗*^	−0.28^*∗*^	−0.18	x	0.19	0.92^*∗*^	0.58^*∗*^	0.48^*∗*^	0.39^*∗*^	0.89^*∗*^	−0.11
K	−0.02	0.26^*∗*^	0.00	−0.10	0.01	−0.03	0.28^*∗*^	−0.12	x	0.12	0.26^*∗*^	−0.05	0.45^*∗*^	0.07	0.08
P	−0.10	−0.20	0.63^*∗*^	0.21^*∗*^	−0.39^*∗*^	−0.39^*∗*^	−0.22^*∗*^	0.89^*∗*^	−0.19	x	0.55^*∗*^	0.47^*∗*^	0.40^*∗*^	0.97^*∗*^	−0.13
Sr	−0.04	−0.06	0.37^*∗*^	0.10	−0.16	0.03	−0.20^*∗*^	0.42^*∗*^	−0.03	0.40^*∗*^	x	0.39^*∗*^	0.36^*∗*^	0.55^*∗*^	−0.08
Ba	0.03	−0.07	0.23^*∗*^	0.01	−0.20	−0.16	−0.13	0.37^*∗*^	−0.17	0.34^*∗*^	0.32^*∗*^	x	0.22^*∗*^	0.43^*∗*^	−0.05
Na	−0.06	−0.10	0.65^*∗*^	0.24^*∗*^	−0.26^*∗*^	−0.26^*∗*^	−0.04	0.79^*∗*^	0.10	0.80^*∗*^	0.41^*∗*^	0.22^*∗*^	x	0.36^*∗*^	−0.10
Ca	−0.08	−0.15	0.62^*∗*^	0.22^*∗*^	−0.36^*∗*^	−0.32^*∗*^	−0.18	0.86^*∗*^	−0.17	0.96^*∗*^	0.38^*∗*^	0.31^*∗*^	0.76^*∗*^	x	−0.11
Age	0.00	0.05	−0.22^*∗*^	0.10	0.17	0.02	0.08	−0.28^*∗*^	0.11	−0.29^*∗*^	−0.30^*∗*^	−0.19	−0.22^*∗*^	−0.29^*∗*^	x
	Neck														

^*∗*^Statistically significant.

**Table 4:** Concentrations of elements (in mg/kg on dry mass basis) and differences between them in the cancellous and cortical bone of the femur according to sex (*N* = 96).

	Femoral head			Femoral neck		
	AM ± SD			AM ± SD		
	Med.			Med.		
	QL–QU			QL–QU		
	Men (*n* = 39)	Women (*n* = 57)	M-W	Men (*n* = 39)	Women (*n* = 57)	M-W
Ca	142503 ± 36420	132738 ± 35772	NS	154831 ± 43196	158841 ± 38764	NS
	139422	122400		153224	156350	
	108142–174294	104630–158906		125302–181456	125986–186928	

P	65624 ± 16587	60738 ± 16755	NS	69124 ± 19596	71697 ± 17421	NS
	65522	55350.5		70672.4	68560	
	50535.9–79484	47526.7–72451		55192.08–82266	57827.5–84699	

Mg	1492.9 ± 344.5	1415.2 ± 368.5	NS	1566.1 ± 345.1	1599.3 ± 304.5	NS
	1533.8	1335.6		1593.8	1606.9	
	1205.1–1784.7	1112.9–1644.6		1283.2–1793.7	1382.9–1790.4	

Na	5522.7 ± 1096.3	5428 ± 1014.7	NS	4617.7 ± 1025.9	4724.7 ± 780.2	NS
	5765.7	5399.5		4628.4	4719	
	4627.7–6308.9	4772.7–5824.5		3717.3–5343.6	4126.5–5223.4	

K	677.3 ± 260.7	955.3 ± 848.3	<0.05	775 ± 791.1	1107 ± 1311.5	<0.01
	584.91	780		570	792.08	
	490–840	570–1049.5		435.64–934.58	600–1168.32	

Zn	76.75 ± 15.37	68.91 ± 15.77	<0.05	69.63 ± 13.37	68.07 ± 13.46	NS
	76.25	64.54		69.52	64.27	
	66.42–89.45	56.53–79.47		58.76–75.45	58.53–76.86	

Cu	1.16 ± 1	0.74 ± 0.75	<0.05	0.97 ± 1.25	0.83 ± 1.09	NS
	1.07	0.64		0.8	0.58	
	0.04–1.77	0.04–1.14		0.04–1.44	0.04–1.1	

Cr	1.34 ± 2.47	1.33 ± 2.1	NS	1.18 ± 1.33	1.62 ± 1.95	NS
	0.61	0.44		0.89	0.76	
	0.12–1.41	0.12–1.69		0.12–1.44	0.12–2.28	

Ni	0.88 ± 1.6	0.41 ± 0.93	<0.05	0.99 ± 2.62	0.64 ± 2.03	NS
	0.21	0.03		0.03	0.03	
	0.03–0.94	0.03–0.39		0.03–0.96	0.03–0.41	

Fe	131.01 ± 114.31	119.91 ± 101.04	NS	111.75 ± 86.48	145.04 ± 165.46	NS
	96.44	89.82		80.77	93.52	
	46.04–178.11	58.98–128.07		50.35–141.99	56.73–164.58	

Sr	45.22 ± 30.76	43.14 ± 23.31	NS	47.24 ± 26.88	47.28 ± 19.58	NS
	39.89	36.29		42.31	44.87	
	26.16–48.57	25.96–50.38		29.27–49.86	33.54–56.15	

Mo	0.44 ± 0.51	0.65 ± 0.64	NS	0.8 ± 0.74	0.69 ± 0.64	NS
	0.18	0.18		0.39	0.18	
	0.18–0.18	0.18–1.32		0.18–1.42	0.18–1.33	

Ba	2.64 ± 1.58	2.35 ± 1.74	NS	2.51 ± 1.24	2.34 ± 1.35	NS
	2.28	1.98		2.47	1.99	
	1.66–3.33	1.34–2.8		1.4–3.49	1.33–3.11	

Pb	1.57 ± 1.7	0.86 ± 1.31	<0.05	1.41 ± 1.65	0.85 ± 1.1	NS
	0.32	0.32		0.32	0.32	
	0.32–2.8	0.32–0.32		0.32–2.12	0.32–1.15	

AM: arithmetic mean; SD: standard deviation; Med.: median; QL: lower quartile; QU: upper quartile; M-W: Mann-Whitney *U*-test; *p*: level of significance; NS: nonsignificant difference.

**Table 5:** Spearman correlation coefficients for metals found in femoral neck by sex.

Neck	Mo	Cr	Zn	Pb	Cu	Ni	Fe	Mg	K	P	Sr	Ba	Na	Ca	Age
	Women														
Mo	x	0.04	−0.11	−0.25	−0.40^*∗*^	−0.06	−0.08	−0.05	−0.13	−0.03	0.08	0.24	−0.09	0.01	−0.15
Cr	0.14	x	0.06	−0.10	0.25	0.37^*∗*^	0.49^*∗*^	0.06	0.16	−0.04	0.00	0.05	0.07	0.00	−0.02
Zn	−0.15	−0.26	x	0.11	0.12	−0.12	−0.13	0.72^*∗*^	0.01	0.56^*∗*^	0.29^*∗*^	0.23	0.57^*∗*^	0.54^*∗*^	−0.22
Pb	−0.44^*∗*^	−0.31	0.43^*∗*^	x	0.15	0.05	−0.08	0.05	−0.08	−0.04	−0.14	−0.15	0.09	−0.03	0.23
Cu	0.00	0.25	−0.42^*∗*^	0.07	x	0.54^*∗*^	0.50^*∗*^	−0.10	0.10	−0.27^*∗*^	−0.03	−0.21	−0.15	−0.25	0.11
Ni	−0.09	0.19	−0.24	−0.09	0.59^*∗*^	x	0.33^*∗*^	−0.19	0.08	−0.30^*∗*^	−0.10	−0.18	−0.15	−0.22	0.00
Fe	0.10	0.22	−0.24	−0.17	0.14	−0.10	x	−0.18	0.27^*∗*^	−0.25	−0.05	−0.16	−0.02	−0.18	0.09
Mg	−0.27	−0.26	0.80^*∗*^	0.59^*∗*^	−0.44^*∗*^	−0.41^*∗*^	−0.17	x	−0.17	0.88^*∗*^	0.35^*∗*^	0.44^*∗*^	0.78^*∗*^	0.83^*∗*^	−0.25
K	0.10	0.43^*∗*^	−0.02	−0.06	−0.02	−0.04	0.27	−0.15	x	−0.26	−0.01	−0.07	0.05	−0.21	−0.11
P	−0.12	−0.35^*∗*^	0.76^*∗*^	0.56^*∗*^	−0.51^*∗*^	−0.49^*∗*^	−0.15	0.91^*∗*^	−0.22	x	0.35^*∗*^	0.42^*∗*^	0.78^*∗*^	0.94^*∗*^	−0.29^*∗*^
Sr	−0.11	−0.11	0.48^*∗*^	0.39^*∗*^	−0.31	0.17	−0.36^*∗*^	0.50^*∗*^	−0.11	0.47^*∗*^	x	0.43^*∗*^	0.38^*∗*^	0.30^*∗*^	−0.32^*∗*^
Ba	−0.21	−0.24	0.24	0.17	−0.20	−0.19	−0.06	0.28	−0.28	0.22	0.19	x	0.34^*∗*^	0.38^*∗*^	−0.19
Na	0.01	−0.25	0.78^*∗*^	0.44^*∗*^	−0.38^*∗*^	−0.39^*∗*^	−0.06	0.81^*∗*^	0.06	0.83^*∗*^	0.48^*∗*^	0.12	x	0.74^*∗*^	−0.24
Ca	−0.12	−0.29	0.79^*∗*^	0.53^*∗*^	−0.48^*∗*^	−0.43^*∗*^	−0.17	0.91^*∗*^	−0.22	0.98^*∗*^	0.49^*∗*^	0.22	0.81^*∗*^	x	−0.25
Age	0.21	0.13	−0.23	0.01	0.32^*∗*^	0.09	0.12	−0.39^*∗*^	0.35^*∗*^	−0.34^*∗*^	−0.31	−0.16	−0.23	−0.40^*∗*^	x
	Men														

^*∗*^Statistically significant.

**Table 6:** Spearman correlation coefficients for metals found in femoral head by sex.

Head	Mo	Cr	Zn	Pb	Cu	Ni	Fe	Mg	K	P	Sr	Ba	Na	Ca	Age
	Women														
Mo	x	−0.13	0.11	0.15	−0.32^*∗*^	0.08	−0.03	0.04	−0.12	0.03	0.00	0.00	−0.28^*∗*^	0.03	−0.07
Cr	0.09	x	−0.09	−0.15	0.39^*∗*^	0.21	0.27^*∗*^	0.06	0.10	0.02	−0.13	0.09	0.24	0.00	0.04
Zn	0.35^*∗*^	−0.04	x	−0.02	−0.07	−0.01	0.14	0.75^*∗*^	0.12	0.78^*∗*^	0.48^*∗*^	0.42^*∗*^	0.42^*∗*^	0.80^*∗*^	−0.13
Pb	−0.05	−0.16	0.03	x	−0.12	0.02	0.01	−0.09	−0.02	−0.06	−0.10	0.00	−0.08	−0.02	0.11
Cu	−0.02	0.57^*∗*^	−0.35^*∗*^	−0.10	x	0.28^*∗*^	0.13	0.04	0.41^*∗*^	−0.06	−0.07	−0.16	0.24	−0.07	0.07
Ni	−0.25	0.20	−0.49^*∗*^	−0.15	0.51^*∗*^	x	−0.14	0.03	−0.22	0.06	0.04	0.05	−0.07	0.07	0.06
Fe	0.20	0.36^*∗*^	0.15	0.08	0.16	−0.25	x	0.02	0.17	0.06	0.03	−0.07	0.06	0.04	0.35^*∗*^
Mg	0.24	0.06	0.85^*∗*^	0.08	−0.26	−0.42^*∗*^	0.10	x	0.21	0.90^*∗*^	0.59^*∗*^	0.54^*∗*^	0.42^*∗*^	0.89^*∗*^	−0.18
K	0.34^*∗*^	0.25	0.35^*∗*^	0.21	0.09	−0.29	0.50^*∗*^	0.33^*∗*^	x	0.12	0.23	0.03	0.46^*∗*^	0.09	0.00
P	0.32^*∗*^	0.05	0.84^*∗*^	−0.07	−0.34^*∗*^	−0.52^*∗*^	0.16	0.93^*∗*^	0.34^*∗*^	x	0.59^*∗*^	0.56^*∗*^	0.43^*∗*^	0.99^*∗*^	−0.20
Sr	0.16	−0.06	0.57^*∗*^	−0.07	−0.07	−0.18	−0.10	0.56^*∗*^	0.44^*∗*^	0.52^*∗*^	x	0.49^*∗*^	0.38^*∗*^	0.58^*∗*^	−0.18
Ba	0.06	−0.05	0.18	0.06	−0.18	0.02	0.00	0.25	−0.05	0.22	0.22	x	0.32^*∗*^	0.53^*∗*^	−0.10
Na	0.10	−0.08	0.38^*∗*^	0.45^*∗*^	−0.25	−0.37^*∗*^	0.21	0.29	0.54^*∗*^	0.31	0.29	0.03	x	0.40^*∗*^	−0.30^*∗*^
Ca	0.18	0.01	0.78^*∗*^	0.02	−0.32^*∗*^	−0.50^*∗*^	0.05	0.87^*∗*^	0.22	0.92^*∗*^	0.52^*∗*^	0.18	0.23	x	−0.19
Age	0.16	0.00	0.03	0.16	0.06	0.07	−0.15	0.06	0.20	0.12	0.12	0.10	0.14	0.17	x
	Men														

^*∗*^Statistically significant.

**Table 7:** Concentrations of elements (in mg/kg on dry mass basis) and differences between them in the cancellous and cortical bone of the femur according to cigarette smoking (*N* = 96).

Metal	Femoral head			Femoral neck		
	AM ± SD			AM ± SD		
	Med.			Med.		
	QL–QU			QL–QU		
	Smokers (*n* = 23)	Nonsmokers (*n* = 73)	M-W	Smokers (*n* = 23)	Nonsmokers (*n* = 73)	M-W
Ca	117495 ± 28846	142758 ± 36287	*p* < 0.01	163117 ± 38410	155351 ± 41149	NS
	109008	139422		146176	153604	
	97994–139194	112362–169574		134048–203900	125302–181456	

P	53711 ± 13014	65562 ± 16893	*p* < 0.01	73176 ± 16717	69857 ± 18783	NS
	50535.9	64623		68560	71192	
	44247–63841	51705–78096		59106–90919	56192–82532	

Mg	1300.7 ± 293.7	1492.8 ± 367.3	*p* < 0.05	1673.5 ± 294.2	1558.2 ± 325	NS
	1288.8	1455.6		1657.2	1552.9	
	1094.1–1521.9	1219.4–1764.8		1400–1934.1	1342.4–1740.4	

Na	5479.5 ± 897.4	5462.4 ± 1091.7	NS	4878.5 ± 874.6	4619.1 ± 884.6	NS
	5383.98	5451.2		4628.4	4663.8	
	4955.94–5915.15	4648.33–6169.32		4196.9–5467.3	3935.5–5246.2	

K	789.2 ± 375.5	859.1 ± 759	NS	964 ± 950.6	974.7 ± 1194.8	NS
	712.9	663.4		637.3	660	
	550–1039.6	519.6–960		534.7–1138.6	500–980	

Zn	66.01 ± 12.24	74.01 ± 16.63	*p* < 0.05	73.67 ± 15.54	67.14 ± 12.33	NS
	65.91	72.1		66.77	65.87	
	55.04–75.8	62.64–89.45		62.63–83.77	57.56–75.2	

Cu	0.94 ± 0.78	0.9 ± 0.91	NS	0.88 ± 1.46	0.89 ± 1.05	NS
	0.89	0.72		0.04	0.79	
	0.04–1.45	0.04–1.5		0.04–1.44	0.04–1.18	

Cr	2 ± 3.57	1.12 ± 1.6	NS	1.65 ± 1.92	1.38 ± 1.68	NS
	0.43	0.5		0.95	0.76	
	0.12–1.95	0.12–1.41		0.45–2.28	0.12–1.58	

Ni	0.72 ± 1.47	0.56 ± 1.19	NS	1.28 ± 3.11	0.63 ± 1.95	NS
	0.03	0.03		0.03	0.03	
	0.03–1.05	0.03–0.69		0.03–1.15	0.03–0.49	

Fe	111.55 ± 79.08	128.47 ± 113.56	NS	94.12 ± 44.24	143.3 ± 156.24	NS
	90.8	96.44		88.64	93.52	
	47.26–171.83	54.54–142.18		56.73–136.87	55.28–167.62	

Sr	38.43 ± 14.88	45.73 ± 29.03	NS	49.82 ± 16.72	46.46 ± 24.32	NS
	36.39	36.74		45.73	41.89	
	25.22–44.43	26.16–52.99		39.73–57.13	29.86–52.54	

Mo	0.54 ± 0.6	0.57 ± 0.6	NS	0.66 ± 0.58	0.75 ± 0.71	NS
	0.18	0.18		0.18	0.18	
	0.18–1.02	0.18–1.15		0.18–1.21	0.18–1.43	

Ba	2.5 ± 2.1	2.5 ± 1.5	NS	2.71 ± 1.54	2.32 ± 1.21	NS
	2.04	2.09		2.63	2.07	
	1.52–2.97	1.46–3.08		1.41–3.48	1.35–3.11	

Pb	1.38 ± 1.67	1.08 ± 1.46	NS	1.04 ± 1.33	1.09 ± 1.4	NS
	0.32	0.32		0.32	0.32	
	0.32–2.18	0.32–1.44		0.32–1.35	0.32–1.57	

AM: arithmetic mean; SD: standard deviation; Med.: median: M-W, QL: lower quartile; QU: upper quartile; M-W: Mann-Whitney *U*-test; *p*: level of significance; NS: nonsignificant difference.

**Table 8:** Spearman correlation coefficients for metals found in femoral neck of smokers and nonsmokers.

	Mo	Cr	Zn	Pb	Cu	Ni	Fe	Mg	K	P	Sr	Ba	Na	Ca	Age
Neck	Nonsmoker														
Mo	x	0.20	−0.14	−0.31^*∗*^	−0.13	0.01	0.06	−0.17	0.00	−0.13	−0.03	0.01	−0.11	−0.10	−0.08
Cr	−0.22	x	−0.05	−0.17	0.23^*∗*^	0.17	0.39^*∗*^	−0.09	0.27^*∗*^	−0.22	−0.10	−0.01	−0.08	−0.16	0.10
Zn	−0.08	−0.25	x	0.29^*∗*^	−0.05	−0.13	−0.21	0.70^*∗*^	0.00	0.61^*∗*^	0.34^*∗*^	0.19	0.63^*∗*^	0.60^*∗*^	−0.22
Pb	−0.32	−0.22	0.15	x	0.09	−0.01	−0.12	0.33^*∗*^	−0.13	0.28^*∗*^	0.05	−0.10	0.29^*∗*^	0.27^*∗*^	0.08
Cu	−0.37	0.43^*∗*^	−0.21	0.23	x	0.51^*∗*^	0.32^*∗*^	−0.26^*∗*^	−0.05	−0.40^*∗*^	−0.25^*∗*^	−0.19	−0.26^*∗*^	−0.39^*∗*^	0.12
Ni	−0.19	0.74^*∗*^	−0.28	0.05	0.69^*∗*^	x	0.15	−0.25^*∗*^	−0.03	−0.39^*∗*^	−0.03	−0.11	−0.25^*∗*^	−0.32^*∗*^	0.05
Fe	−0.39	0.35	−0.03	−0.19	0.40	0.17	x	−0.24^*∗*^	0.26^*∗*^	−0.27^*∗*^	−0.20	−0.07	−0.06	−0.24^*∗*^	0.02
Mg	−0.08	−0.21	0.89^*∗*^	0.11	−0.14	−0.39	0.08	x	−0.16	0.90^*∗*^	0.45^*∗*^	0.37^*∗*^	0.82^*∗*^	0.86^*∗*^	−0.30^*∗*^
K	−0.08	0.18	−0.08	−0.01	0.12	−0.11	0.31	0.02	x	−0.23^*∗*^	−0.03	−0.20	0.02	−0.22	0.09
P	0.02	−0.21	0.71^*∗*^	−0.02	−0.30	−0.38	0.07	0.86^*∗*^	−0.13	x	0.42^*∗*^	0.36^*∗*^	0.82^*∗*^	0.95^*∗*^	−0.34^*∗*^
Sr	−0.14	−0.03	0.35	0.48^*∗*^	0.17	0.18	−0.18	0.31	−0.13	0.27	x	0.35^*∗*^	0.41^*∗*^	0.39^*∗*^	−0.31^*∗*^
Ba	0.19	−0.39	0.28	0.34	−0.19	−0.38	−0.31	0.35	−0.09	0.31	0.11	x	0.25^*∗*^	0.33^*∗*^	−0.20
Na	0.14	−0.26	0.65^*∗*^	0.08	−0.21	−0.34	0.11	0.69^*∗*^	0.31	0.66^*∗*^	0.35	0.12	x	0.78^*∗*^	−0.28^*∗*^
Ca	0.00	−0.19	0.69^*∗*^	0.00	−0.30	−0.35	0.08	0.84^*∗*^	−0.05	0.97^*∗*^	0.26	0.28	0.63^*∗*^	x	−0.34^*∗*^
Age	0.26	0.09	−0.07	−0.06	0.33	0.08	0.38	−0.06	0.23	−0.18	−0.06	−0.18	0.11	−0.17	x
	Smoker														

^*∗*^Statistically significant.

**Table 9:** Spearman correlation coefficients for metals found in femoral head of smokers and nonsmokers.

Head	Mo	Cr	Zn	Pb	Cu	Ni	Fe	Mg	K	P	Sr	Ba	Na	Ca	Age
	Nonsmoker														
Mo	x	−0.11	0.12	0.13	−0.23^*∗*^	−0.16	0.08	0.04	0.19	0.11	0.08	−0.03	−0.04	0.05	0.03
Cr	−0.01	x	0.04	−0.08	0.43^*∗*^	0.15	0.28^*∗*^	0.10	0.13	0.06	−0.04	0.10	0.17	0.05	0.05
Zn	0.04	−0.32	x	0.04	−0.04	−0.16	0.17	0.79^*∗*^	0.20	0.81^*∗*^	0.52^*∗*^	0.38^*∗*^	0.50^*∗*^	0.77^*∗*^	−0.15
Pb	−0.29	−0.37	0.33	x	0.01	0.07	0.06	0.12	0.01	0.09	−0.09	0.00	0.13	0.13	0.20
Cu	−0.17	0.47^*∗*^	−0.35	−0.15	x	0.40^*∗*^	0.15	0.00	0.15	−0.11	−0.08	−0.09	0.00	−0.12	0.05
Ni	0.07	0.20	−0.07	−0.26	0.53^*∗*^	x	−0.22	−0.14	−0.37^*∗*^	−0.18	−0.13	0.04	−0.32^*∗*^	−0.19	−0.01
Fe	−0.04	0.55^*∗*^	−0.03	−0.10	0.16	−0.05	x	0.05	0.33^*∗*^	0.11	0.01	−0.08	0.22	0.05	0.14
Mg	0.16	−0.04	0.75^*∗*^	0.00	−0.27	−0.21	0.08	x	0.25^*∗*^	0.91^*∗*^	0.61^*∗*^	0.49^*∗*^	0.49^*∗*^	0.88^*∗*^	−0.21
K	−0.30	0.30	−0.19	0.04	0.39	−0.05	0.18	0.05	x	0.19	0.29^*∗*^	0.01	0.54^*∗*^	0.13	0.07
P	−0.07	0.00	0.73^*∗*^	−0.10	−0.36	−0.19	0.07	0.85^*∗*^	−0.08	x	0.6^*∗*^	0.48^*∗*^	0.52^*∗*^	0.95^*∗*^	−0.27^*∗*^
Sr	−0.09	−0.35	0.47^*∗*^	0.10	−0.14	0.16	−0.28	0.36	0.13	0.36	x	0.38^*∗*^	0.38^*∗*^	0.59^*∗*^	−0.12
Ba	0.03	0.03	0.57^*∗*^	0.23	−0.27	0.05	0.07	0.49^*∗*^	−0.26	0.56^*∗*^	0.48^*∗*^	x	0.23	0.44^*∗*^	−0.08
Na	−0.48^*∗*^	−0.02	0.28	0.45^*∗*^	0.13	0.15	−0.23	−0.02	0.13	0.07	0.11	0.14	x	0.46^*∗*^	−0.12
Ca	−0.10	−0.08	0.75^*∗*^	−0.02	−0.29	−0.16	0.02	0.83^*∗*^	−0.07	0.97^*∗*^	0.38	0.54^*∗*^	0.07	x	−0.23^*∗*^
Age	0.13	−0.07	−0.25	−0.16	0.10	0.21	−0.13	−0.11	0.03	−0.19	−0.09	−0.08	−0.13	−0.16	x
	Smoker														

^*∗*^Statistically significant.

**Table 10:** Concentrations of elements (in mg/kg on dry mass basis) and differences between them in the cancellous and cortical bone of the femur according to contact with chemicals (*N* = 96).

Contact	Femoral head			Femoral neck		
	AM ± SD			AM ± SD		
	Med.			Med.		
	QL–QU			QL–QU		
	Yes *n* = 29	No *n* = 67	M-W	Yes *n* = 29	No *n* = 67	M-W
Ca	129760 ± 30767	139711 ± 38092	NS	155742 ± 38132	157848 ± 41674	NS
	121696	133780		153604	150986	
	106560–143042	106146–173244		131382–181456	125924–184734	

Mg	1385.5 ± 294.8	1473.3 ± 382.7	NS	1559.1 ± 338.4	1597.4 ± 314	NS
	1334.5	1384.04		1522	1612.97	
	1183.17–1610.5	1159.13–1784.71		1387.55–1732.5	1372.67–1793.73	

P	60891.1 ± 15375.9	63515.9 ± 17395.5	NS	68897.5 ± 17470.4	71411.8 ± 18695.6	NS
	59071.29	59118		70672.38	68560	
	48262–69132.67	47872.9–78380.2		55192.08–81000	57827.45–84699.01	

Na	5427.4 ± 1079.4	5483.5 ± 1036.1	NS	4559.5 ± 998.3	4733.9 ± 833.2	NS
	5334.02	5456.31		4458	4812.28	
	4975.84–6217.92	4648.33–6093.08		3935.54–5181.37	4072.3–5358.02	

K	687.4 ± 251.4	909.4 ± 796.7	NS	1094.3 ± 1822.1	919.2 ± 663.7	NS
	611.65	712.87		570	770	
	500–810	564.36–1039.6		450–950	534.65–1138.61	

Zn	72.8 ± 13.5	71.8 ± 17.1	NS	69.4 ± 14.6	68.4 ± 12.9	NS
	71.01	69.75		69.42	65.4	
	62.61–81.98	57.65–87.98		57.56–76.51	58.76–75.45	

Cu	1 ± 1	0.9 ± 0.8	NS	1 ± 1.5	0.8 ± 1	NS
	0.9	0.76		0.58	0.77	
	0.04–1.66	0.04–1.37		0.04–1.22	0.04–1.12	

Cr	1.6 ± 3.1	1.2 ± 1.8	NS	1.6 ± 1.8	1.4 ± 1.7	NS
	0.5	0.48		0.95	0.7	
	0.12–1.68	0.12–1.67		0.51–1.91	0.12–1.7	

Ni	1.1 ± 1.9	0.4 ± 0.8	*p* < 0.05	1.2 ± 3	0.6 ± 1.9	NS
	0.21	0.03		0.03	0.03	
	0.03–1.08	0.03–0.5		0.03–0.54	0.03–0.49	

Fe	119.2 ± 104.9	126.7 ± 107.4	NS	132.7 ± 119.1	131 ± 148	NS
	96.44	90.8		91.7	90.24	
	47.26–144.48	55.58–142.18		71.23–141.99	50.35–155.29	

Sr	39.3 ± 26.7	46 ± 26.3	NS	44.4 ± 23.5	48.5 ± 22.4	NS
	34.17	37.97		42.55	44.54	
	25.81–41.54	26.96–54.91		29.8–47.52	33.27–56.27	

Mo	0.5 ± 0.6	0.6 ± 0.6	NS	0.8 ± 0.7	0.7 ± 0.7	NS
	0.18	0.18		0.86	0.18	
	0.18–0.18	0.18–1.15		0.18–1.44	0.18–1.33	

Ba	2.3 ± 1.3	2.5 ± 1.8	NS	2.5 ± 1.3	2.4 ± 1.3	NS
	1.9	2.1		2.25	2.24	
	1.57–2.94	1.45–3.09		1.46–3.49	1.38–3.11	

Pb	1.4 ± 1.8	1 ± 1.4	NS	1 ± 1.3	1.1 ± 1.4	NS
	0.32	0.32		0.32	0.32	
	0.32–2.18	0.32–1.53		0.32–1.28	0.32–1.81	

AM: arithmetic mean; SD: standard deviation; Med.: median; QL: lower quartile; QU: upper quartile; M-W, Mann-Whitney *U*-test; *p*: level of significance; NS: nonsignificant difference.

**Table 11:** Spearman correlation coefficients for metals found in femoral neck of patients with chemicals contact and without contact.

Neck	Mo	Cr	Zn	Pb	Cu	Ni	Fe	Mg	K	P	Sr	Ba	Na	Ca	Age
Contact with chemicals	No														
Mo	x	−0.17	0.16	−0.01	−0.27^*∗*^	−0.09	−0.04	0.10	0.00	0.13	0.10	−0.03	−0.20	0.12	0.00
Cr	0.14	x	−0.08	−0.05	0.42^*∗*^	0.15	0.32^*∗*^	0.01	0.22	0.02	−0.07	0.02	0.20	0.00	0.13
Zn	−0.07	0.11	x	0.19	−0.11	−0.16	0.13	0.81^*∗*^	0.12	0.81^*∗*^	0.54^*∗*^	0.49^*∗*^	0.52^*∗*^	0.81^*∗*^	−0.09
Pb	0.08	−0.38^*∗*^	−0.20	x	0.05	0.12	−0.07	0.27^*∗*^	−0.07	0.22	−0.01	0.19	0.14	0.25^*∗*^	0.03
Cu	−0.13	0.47^*∗*^	−0.06	−0.16	x	0.36^*∗*^	0.21	−0.12	0.32^*∗*^	−0.21	−0.17	−0.20	0.17	−0.21	0.09
Ni	−0.09	0.21	−0.16	−0.25	0.59^*∗*^	x	−0.13	−0.16	−0.23	−0.17	−0.12	−0.03	−0.06	−0.15	0.07
Fe	0.25	0.36	0.16	0.22	0.04	−0.28	x	0.02	0.30^*∗*^	0.06	0.11	−0.05	0.08	0.05	0.21
Mg	−0.08	0.21	0.81^*∗*^	−0.43^*∗*^	0.11	−0.04	0.04	x	0.21	0.92^*∗*^	0.59^*∗*^	0.57^*∗*^	0.50^*∗*^	0.91^*∗*^	−0.20
K	0.25	0.06	0.08	0.29	−0.03	−0.34	0.28	0.01	x	0.10	0.29^*∗*^	−0.01	0.41^*∗*^	0.07	−0.06
P	−0.09	0.17	0.78^*∗*^	−0.43^*∗*^	0.09	−0.05	0.16	0.87^*∗*^	0.09	x	0.62^*∗*^	0.61^*∗*^	0.51^*∗*^	0.99^*∗*^	−0.23
Sr	−0.04	−0.21	0.47^*∗*^	−0.11	0.11	0.16	−0.40^*∗*^	0.51^*∗*^	0.09	0.29	x	0.49^*∗*^	0.44^*∗*^	0.61^*∗*^	−0.21
Ba	0.03	0.18	0.19	−0.27	0.05	0.20	0.06	0.30	−0.21	0.09	0.16	x	0.35^*∗*^	0.61^*∗*^	−0.03
Na	0.02	0.00	0.33	0.37	−0.20	−0.42^*∗*^	0.23	0.10	0.54^*∗*^	0.14	0.20	−0.16	x	0.49^*∗*^	−0.21
Ca	−0.25	0.08	0.72^*∗*^	−0.39^*∗*^	0.06	−0.04	−0.04	0.76^*∗*^	−0.05	0.90^*∗*^	0.32	−0.07	0.00	x	−0.23
Age	0.15	0.03	−0.36	0.24	0.07	0.08	−0.05	−0.36	0.34	−0.21	−0.30	−0.22	0.00	−0.22	x
	Yes														

^*∗*^Statistically significant.

**Table 12:** Spearman correlation coefficients for metals found in femoral head of patients with chemicals contact and without contact.

Head	Mo	Cr	Zn	Pb	Cu	Ni	Fe	Mg	K	P	Sr	Ba	Na	Ca	Age
Contact with chemicals	No														
Mo	x	0.04	−0.07	−0.25^*∗*^	−0.17	−0.06	−0.15	−0.08	−0.06	−0.06	0.04	0.16	−0.09	−0.08	0.00
Cr	0.29	x	−0.06	−0.18	0.34^*∗*^	0.36^*∗*^	0.43^*∗*^	0.00	0.30^*∗*^	−0.14	0.01	0.00	0.02	−0.15	0.03
Zn	−0.30	−0.13	x	0.16	−0.05	−0.10	−0.21	0.71^*∗*^	−0.09	0.60^*∗*^	0.34^*∗*^	0.22	0.59^*∗*^	0.62^*∗*^	−0.14
Pb	−0.46^*∗*^	−0.23	0.46^*∗*^	x	0.07	0.05	−0.06	0.16	−0.20	0.11	−0.01	0.00	0.18	0.14	0.10
Cu	−0.11	0.06	−0.19	0.25	x	0.61^*∗*^	0.42^*∗*^	−0.23	0.06	−0.40^*∗*^	−0.15	−0.17	−0.28^*∗*^	−0.38^*∗*^	0.14
Ni	−0.01	0.13	−0.25	−0.04	0.51^*∗*^	x	0.21	−0.24	0.05	−0.35^*∗*^	−0.04	−0.16	−0.19	−0.31^*∗*^	0.04
Fe	0.28	0.20	−0.12	−0.23	0.16	−0.05	x	−0.20	0.33^*∗*^	−0.27^*∗*^	−0.22	−0.14	−0.06	−0.26^*∗*^	−0.02
Mg	−0.30	−0.33	0.81^*∗*^	0.49^*∗*^	−0.32	−0.37^*∗*^	−0.17	x	−0.16	0.88^*∗*^	0.36^*∗*^	0.37^*∗*^	0.78^*∗*^	0.88^*∗*^	−0.27^*∗*^
K	0.09	0.34	0.16	0.07	−0.01	−0.07	0.17	−0.03	x	−0.24^*∗*^	−0.08	−0.07	0.03	−0.24	−0.06
P	−0.18	−0.36	0.70^*∗*^	0.43^*∗*^	−0.38^*∗*^	−0.50^*∗*^	−0.07	0.91^*∗*^	−0.16	x	0.37^*∗*^	0.34^*∗*^	0.78^*∗*^	0.99^*∗*^	−0.35^*∗*^
Sr	−0.18	−0.22	0.42^*∗*^	0.35	−0.21	0.21	−0.19	0.56^*∗*^	0.04	0.48^*∗*^	x	0.39^*∗*^	0.44^*∗*^	0.36^*∗*^	−0.37^*∗*^
Ba	−0.26	−0.31	0.19	0.03	−0.26	−0.18	−0.10	0.38^*∗*^	−0.31	0.35	0.19	x	0.31^*∗*^	0.33^*∗*^	−0.17
Na	0.02	−0.33	0.75^*∗*^	0.37^*∗*^	−0.25	−0.41^*∗*^	0.02	0.82^*∗*^	0.09	0.84^*∗*^	0.36	0.09	x	0.79^*∗*^	−0.30^*∗*^
Ca	−0.14	−0.19	0.62^*∗*^	0.43^*∗*^	−0.29	−0.30	0.02	0.79^*∗*^	−0.06	0.86^*∗*^	0.46^*∗*^	0.21	0.71^*∗*^	x	−0.37^*∗*^
Age	−0.02	−0.03	−0.25	0.09	0.35	−0.03	0.15	−0.21	0.15	−0.07	−0.36	−0.05	−0.12	0.03	x
	Yes														

^*∗*^Statistically significant.

**Table 13:** Concentrations of elements (in mg/kg on dry mass basis) and differences between them in the cancellous and cortical bone of the femur according to alcohol consumption (*N* = 96).

Alcohol	Femoral head			Femoral neck		
	AM ± SD			AM ± SD		
	Med.			Med.		
	QL–QU			QL–QU		
	No *n* = 46	Yes *n* = 50	M-W	No *n* = 46	Yes *n* = 50	M-W
Ca	136442.4 ± 34544.3	136947.8 ± 37950.4	NS	161328.5 ± 39085.7	153425.4 ± 41695.1	NS
	129183	133763		159462	146143	
	108960–160694	102876–162084		128094–187160	122380–181456	

P	62247.9 ± 16107.8	63160 ± 17517	NS	72665.4 ± 17803.5	68800.2 ± 18692.6	NS
	58510.4	61377.6		71514.5	67434.4	
	49736–76200	47466–75652.4		58714–85978	55156.4–82266	

Mg	1436.1 ± 379.2	1456.6 ± 343.3	NS	1581.4 ± 297.8	1589.8 ± 342.6	NS
	1340.9	1442.1		1622	1566.4	
	1159.1–1734	1183.2–1678.6		1372.7–1740.4	1376.9–1853.8	

Na	5347.8 ± 1009.6	5575.7 ± 1073	NS	4792.8 ± 763.9	4578.6 ± 979.2	NS
	5315	5649.5		4862.9	4507.8	
	4627.7–5916.2	4955.9–6308.9		4211–5295.8	3747.7–5282.2	

K	942.1 ± 917	750.6 ± 348.2	NS	953.4 ± 728.9	989.4 ± 1419.4	NS
	694.3	657.1 5		810.3	603	
	564.4–1049.5	04.9–844.7		547.2–1090	450–960	

Zn	70.02 ± 15.65	74 ± 16.25	NS	66.99 ± 11.9	70.28 ± 14.54	NS
	65.58	75.42		64.82	66.66	
	57.68–79.47	60.27–85.83		58.21–73.3	59.09–76.86	

Cu	0.68 ± 0.76	1.12 ± 0.93	<0.05	0.6 ± 0.75	1.15 ± 1.38	<0.05
	0.52	1.06		0.04	0.81	
	0.04–1.14	0.04–1.75		0.04–1.03	0.04–1.44	

Cr	1.2 ± 1.87	1.45 ± 2.56	NS	1.47 ± 1.76	1.41 ± 1.72	NS
	0.44	0.5		0.73	0.84	
	0.12–1.67	0.12–1.68		0.12–1.74	0.12–1.58	

Ni	0.41 ± 0.94	0.78 ± 1.48	<0.05	0.32 ± 0.66	1.21 ± 3.05	NS
	0.03	0.03		0.03	0.03	
	0.03–0.39	0.03–0.89		0.03–0.41	0.03–0.64	

Fe	134.38 ± 115.12	115.25 ± 97.52	NS	136.54 ± 147.76	126.9 ± 132.35	NS
	94.96	90.71		89.54	95.22	
	58.98–146.02	50.43–131.18		56.15–164.75	55.28–152.27	

Sr	45.68 ± 29.49	42.42 ± 23.52	NS	48.05 ± 22.78	46.55 ± 22.82	NS
	35.75	38.31		42.74	43.62	
	25.22–50.5	29.03–48.57		33.27–53.26	30.53–56.15	

Mo	0.7 ± 0.63	0.44 ± 0.55	<0.05	0.77 ± 0.74	0.7 ± 0.62	NS
	0.18	0.18		0.18	0.27	
	0.18–1.32	0.18–0.18		0.18–1.43	0.18–1.33	

Ba	2.3 ± 1.46	2.63 ± 1.85	NS	2.25 ± 1.17	2.56 ± 1.41	NS
	2.01	2.1		1.98	2.42	
	1.34–2.82	1.62–3.15		1.29–3.05	1.42–3.49	

Pb	0.78 ± 1.17	1.49 ± 1.71	<0.05	0.84 ± 1.04	1.29 ± 1.6	NS
	0.32	0.32		0.32	0.32	
	0.32–0.32	0.32–2.71		0.32–1.17	0.32–1.99	

AM: arithmetic mean; SD: standard deviation; Med: median; QL: lower quartile; QU: upper quartile; M-W: Mann-Whitney *U*-test; *p*: level of significance; NS: nonsignificant difference.

**Table 14:** Spearman correlation coefficients for metals found in femoral neck of alcohol drinkers and abstainers.

Neck	Mo	Cr	Zn	Pb	Cu	Ni	Fe	Mg	K	P	Sr	Ba	Na	Ca	Age
Alcohol	Yes														
Mo	x	−0.02	−0.10	−0.37^*∗*^	−0.12	−0.11	−0.06	−0.26	0.02	−0.11	−0.14	−0.06	0.00	−0.13	0.02
Cr	0.23	x	−0.13	−0.30^*∗*^	0.27	0.35^*∗*^	0.26	−0.19	0.26	−0.32^*∗*^	−0.11	−0.30^*∗*^	−0.22	−0.31^*∗*^	−0.02
Zn	−0.17	0.03	x	0.40^*∗*^	−0.41^*∗*^	−0.31^*∗*^	−0.11	0.82^*∗*^	0.04	0.75^*∗*^	0.42^*∗*^	0.24	0.77^*∗*^	0.75^*∗*^	−0.08
Pb	−0.27	−0.05	0.07	x	−0.10	−0.21	−0.18	0.50^*∗*^	0.00	0.48^*∗*^	0.32^*∗*^	0.21	0.41^*∗*^	0.48^*∗*^	−0.03
Cu	−0.22	0.28	0.14	0.35^*∗*^	x	0.57^*∗*^	0.20	−0.36^*∗*^	−0.09	−0.42^*∗*^	−0.12	−0.22	−0.37^*∗*^	−0.41^*∗*^	0.26
Ni	0.07	0.26	0.00	0.28	0.56^*∗*^	x	−0.05	−0.41^*∗*^	−0.14	−0.45^*∗*^	0.16	−0.31^*∗*^	−0.39^*∗*^	−0.41^*∗*^	−0.03
Fe	0.03	0.48^*∗*^	−0.21	−0.03	0.53^*∗*^	0.39^*∗*^	x	−0.01	0.18	−0.05	−0.17	−0.03	0.08	−0.07	−0.01
Mg	−0.05	0.04	0.65^*∗*^	−0.04	−0.19	−0.11	−0.37^*∗*^	x	−0.08	0.91^*∗*^	0.48^*∗*^	0.38^*∗*^	0.81^*∗*^	0.90^*∗*^	−0.11
K	−0.09	0.32^*∗*^	−0.05	−0.20	0.26	0.18	0.41^*∗*^	−0.22	x	−0.18	−0.01	−0.28	0.07	−0.18	0.20
P	−0.10	−0.05	0.51^*∗*^	−0.10	−0.33^*∗*^	−0.29^*∗*^	−0.41^*∗*^	0.88^*∗*^	−0.32^*∗*^	x	0.50^*∗*^	0.38^*∗*^	0.85^*∗*^	0.98^*∗*^	−0.11
Sr	0.06	0.00	0.30^*∗*^	−0.21	−0.24	−0.16	−0.25	0.35^*∗*^	−0.12	0.29	x	0.20	0.44^*∗*^	0.53^*∗*^	−0.26
Ba	0.14	0.19	0.20	−0.32^*∗*^	−0.25	−0.03	−0.23	0.34^*∗*^	−0.02	0.33^*∗*^	0.46^*∗*^	x	0.22	0.39^*∗*^	−0.26
Na	−0.14	0.09	0.50^*∗*^	0.01	−0.12	−0.06	−0.17	0.77^*∗*^	0.00	0.74^*∗*^	0.39^*∗*^	0.28	x	0.82^*∗*^	−0.07
Ca	−0.03	0.04	0.50^*∗*^	−0.10	−0.30^*∗*^	−0.18	−0.31^*∗*^	0.82^*∗*^	−0.28	0.92^*∗*^	0.21	0.27	0.69^*∗*^	x	−0.13
Age	−0.01	−0.04	−0.20	0.31^*∗*^	0.17	0.04	0.00	−0.36^*∗*^	−0.10	−0.40^*∗*^	−0.34^*∗*^	−0.02	−0.39^*∗*^	−0.38^*∗*^	x
	No														

^*∗*^Statistically significant.

**Table 15:** Spearman correlation coefficients for metals found in femoral head of alcohol drinkers and abstainers.

Head	Mo	Cr	Zn	Pb	Cu	Ni	Fe	Mg	K	P	Sr	Ba	Na	Ca	Age
Alcohol	Yes														
Mo	x	0.14	0.15	0.02	0.01	−0.05	0.28^*∗*^	0.07	0.17	0.05	−0.04	0.00	−0.03	−0.06	0.14
Cr	−0.26	x	0.00	−0.32^*∗*^	0.34^*∗*^	0.12	0.28^*∗*^	0.09	0.10	0.09	−0.04	−0.07	0.05	0.08	0.00
Zn	0.14	−0.10	x	0.06	−0.28	−0.33^*∗*^	0.16	0.86^*∗*^	0.14	0.87^*∗*^	0.59^*∗*^	0.38^*∗*^	0.38^*∗*^	0.84^*∗*^	−0.24
Pb	0.17	0.00	0.00	x	−0.08	−0.17	0.01	0.02	0.11	−0.04	−0.07	0.02	0.30^*∗*^	0.03	0.13
Cu	−0.39^*∗*^	0.53^*∗*^	0.01	−0.13	x	0.51^*∗*^	0.08	−0.24	0.14	−0.31^*∗*^	−0.16	−0.29^*∗*^	−0.30^*∗*^	−0.29^*∗*^	0.08
Ni	−0.04	0.24	0.04	0.14	0.25	x	−0.23	−0.31^*∗*^	−0.33^*∗*^	−0.36^*∗*^	−0.17	0.04	−0.36^*∗*^	−0.36^*∗*^	0.11
Fe	−0.19	0.41^*∗*^	0.14	0.09	0.29^*∗*^	−0.06	x	0.10	0.28^*∗*^	0.19	−0.09	0.01	0.15	0.08	−0.11
Mg	0.08	0.04	0.72^*∗*^	0.05	0.12	0.05	−0.02	x	0.17	0.91^*∗*^	0.57^*∗*^	0.41^*∗*^	0.26	0.88^*∗*^	−0.17
K	−0.07	0.26	0.12	−0.07	0.36^*∗*^	−0.16	0.31^*∗*^	0.24	x	0.08	0.29^*∗*^	−0.19	0.39^*∗*^	0.00	0.08
P	0.11	−0.02	0.75^*∗*^	0.08	0.04	0.11	0.02	0.91^*∗*^	0.17	x	0.53^*∗*^	0.44^*∗*^	0.36^*∗*^	0.95^*∗*^	−0.15
Sr	0.11	−0.12	0.48^*∗*^	−0.06	−0.03	0.02	0.04	0.60^*∗*^	0.27	0.60^*∗*^	x	0.28^*∗*^	0.30^*∗*^	0.55^*∗*^	−0.12
Ba	0.03	0.21	0.34^*∗*^	0.04	0.02	0.02	−0.04	0.55^*∗*^	0.16	0.49^*∗*^	0.53^*∗*^	x	0.09	0.41^*∗*^	−0.20
Na	−0.22	0.17	0.39^*∗*^	−0.08	0.36^*∗*^	−0.03	0.12	0.48^*∗*^	0.58^*∗*^	0.44^*∗*^	0.44^*∗*^	0.35^*∗*^	x	0.32^*∗*^	0.00
Ca	0.12	−0.03	0.76^*∗*^	0.10	0.04	0.13	0.02	0.90^*∗*^	0.15	0.99^*∗*^	0.59^*∗*^	0.46^*∗*^	0.39^*∗*^	x	−0.11
Age	−0.08	0.21	−0.09	0.05	0.11	−0.11	0.36^*∗*^	−0.23	0.07	−0.28	−0.16	0.06	−0.20	−0.26	x
	No														

^*∗*^Statistically significant.

**Table 16:** Concentrations of elements (in mg/kg on dry mass basis) and differences between them in the cancellous and cortical bone of the femur in according place of residence (*N* = 96).

	Femoral head			Femoral neck		
	AM ± SD			AM ± SD		
	Med.			Med.		
	QL–QU			QL–QU		
	Villages *n* = 24	Cities *n* = 72	M-W	Villages *n* = 24	Cities *n* = 72	M-W
Ca	133711.4 ± 36808.1	137703.7 ± 36158.5	NS	165008.1 ± 51281.2	154613.7 ± 36191.7	NS
	120389	137906		150816	153269	
	101795–166517	108575–159925		125282–215620	128031–175956	

P	61471.6 ± 17252.3	63140.1 ± 16714.3	NS	74568.7 ± 22839.8	69346.8 ± 16467.5	NS
	55552.4	60363.1		67228.7	69616.2	
	46500.6–77052	49964–75566.2		57776.1–98764.2	57515–78448	

Mg	1391.2 ± 332.4	1465.3 ± 368	NS	1579.8 ± 362.9	1587.8 ± 307.5	NS
	1325.7	1385.8		1583.3	1595.3	
	1103.1–1585.4	1202.8–1761.1		1348.2–1781.9	1385.2–1792.1	

Na	5467.9 ± 1128	5466.1 ± 1022.8	NS	4660.3 ± 904.9	4688.2 ± 884.1	NS
	5461.7	5428.6		4845.9	4642.6	
	4790.7–6362.5	4700.2–6086		4121.8–5297.7	4020.9–5289	

K	1002.9 ± 1178.6	788.9 ± 409.2	NS	835.1 ± 505.6	1017.8 ± 1279.2	NS
	711.4	657.1		629	655	
	534.7–892.7	522.2–971.1		482.3–1049.7	502.4–995	

Zn	69.97 ± 16.17	72.8 ± 16	NS	70.05 ± 17.61	68.25 ± 11.75	NS
	67.13	71.01		66.55	66.21	
	59.73–79.74	60.17–86.07		57.08–81.27	60.18–75.31	

Cu	0.8 ± 0.83	0.95 ± 0.9	NS	1.03 ± 1.45	0.84 ± 1.04	NS
	0.73	0.87		0.75	0.69	
	0.04–1.06	0.04–1.55		0.04–1.3	0.04–1.15	

Cr	1.11 ± 1.36	1.41 ± 2.47	NS	0.82 ± 0.83	1.65 ± 1.9	NS
	0.47	0.49		0.66	0.85	
	0.12–1.69	0.12–1.59		0.12–1.17	0.24–2.32	

Ni	0.68 ± 1.68	0.57 ± 1.1	NS	0.93 ± 3.01	0.74 ± 2	NS
	0.03	0.03		0.03	0.03	
	0.03–0.44	0.03–0.74		0.03–0.46	0.03–0.52	

Fe	141.86 ± 119.1	118.6 ± 101.75	NS	105.95 ± 74.64	140.04 ± 154.46	NS
	123.51	90.71		84.44	96.14	
	50.45–182.49	55.34–123.84		42.47–173.09	63.75–144.17	

Sr	44.13 ± 27.28	43.93 ± 26.37	NS	50.49 ± 23.18	46.19 ± 22.59	NS
	36.75	36.34		52.22	42.39	
	24.16–59.66	26.56–47.53		29.18–62.26	32.35–49.08	

Mo	0.54 ± 0.59	0.57 ± 0.61	NS	0.55 ± 0.56	0.79 ± 0.71	NS
	0.18	0.18		0.18	0.36	
	0.18–1.24	0.18–1.12		0.18–1.15	0.18–1.41	

Ba	2.3 ± 1.57	2.53 ± 1.72	NS	2.24 ± 1.15	2.47 ± 1.35	NS
	1.98	2.09		2.06	2.28	
	1.41–2.56	1.46–3.14		1.32–2.71	1.41–3.35	

Pb	1.03 ± 1.67	1.19 ± 1.47	NS	1.16 ± 1.34	1.05 ± 1.39	NS
	0.32	0.32		0.32	0.32	
	0.32–0.32	0.32–2.01		0.32–1.9	0.32–1.32	

AM: arithmetic mean; SD: standard deviation; Med.: median; QL: lower quartile; QU: upper quartile; M-W, Mann-Whitney *U*-test; *p*: level of significance; NS: nonsignificant difference.

**Table 17:** Concentrations of elements (in mg/kg on dry mass basis) and differences between them in the cancellous and cortical bone of the femur according to age (*N* = 96).

	Age group				
	AM ± SD				
	Med.				
	QL–QU				
	20–50	51–60	61–70	71–80	>80
	*n* = 11	*n* = 27	*n* = 30	*n* = 18	*n* = 10
Ca FH^*∗*^	158198.7 ± 38763.7	128491.7 ± 32287.9	142865.8 ± 41041.9	136323.9 ± 30232.2	117447.4 ± 25831.3
	158656	119666	136459	135038	121188
	119568–194996	104066–158906	104148–180870	112362–158708	92534–138612

P FH	72958.8 ± 17399.0	59432.8 ± 15271.7	65732 ± 18749.4	61975.2 ± 14169.0	52665.89 ± 11992.4
	71803.96	54516.5	63332	62014.79	53548.95
	56613.8–92310.8	48103.8–72450.9	47194–81518	50192.0–73190.1	40807.6–59649.0

Mg FH	1675.5 ± 264.4	1357.3 ± 311.6	1511.3 ± 382.9	1452.2 ± 379.6	1232.7 ± 325.1
	1734.02	1224.56	1481.88	1350.05	1299.93
	1521.88–1919.11	1112.9–1607.55	1200.59–1784.71	1261.19–1635.77	912.79–1380.28

Sr FH	57.38 ± 44.36	38.93 ± 13.78	51.54 ± 32.58	36.22 ± 14.79	34.16 ± 10.28
	42	40.48	36.34	34.31	33.41
	24.48–87.53	24.83–46.18	29.07–60.9	25.47–42.93	26.96–43.55

Ca FN^*∗∗*^	168736.1 ± 46354.7	164040.1 ± 33363.7	163121.1 ± 45040.8	149062.8 ± 37371.2	123043.4 ± 25357.7
	176572	162990	162856	152295	127221
	125302–214280	134490–200640	128094–209680	121488–174288	101530–141492

P FN	77115.9 ± 21627.3	73381.2 ± 14886.5	73111.8 ± 19676.9	66838.4 ± 17941.8	55660.2 ± 11790.0
	82532.67	71312.87	72363.86	67434.43	57634.75
	54786.4–101807.6	60465.3–85978	57948.0–88971.4	55192.08–78304	45022–64054.72

Mg FN	1636.91 ± 307.05	1660.16 ± 272.33	1631.32 ± 358.02	1501.81 ± 305.75	1343.52 ± 260.56
	1544.1	1681.6	1654.69	1507.45	1397.65
	1410.69–1910.4	1478.3–1793.73	1372.67–1865.9	1283.17–1642.48	1175.38–1534.37

Sr FN	60.53 ± 35.13	49.33 ± 21.89	50.53 ± 22.6	36.52 ± 12.22	36.64 ± 11.51
	52.54	44.62	44.97	37.96	34.37
	31.68–80.56	33.76–57.13	34.22–62.84	27.18–44.08	25.64–44.54

^*∗*^FH: femoral head; ^*∗∗*^FN: femoral neck; AM: arithmetic mean; SD: standard deviation; Med.: median; QL: lower quartile; QU: upper quartile.
